# Intravenous iron in patients with heart failure and iron deficiency: an updated meta‐analysis

**DOI:** 10.1002/ejhf.2810

**Published:** 2023-03-08

**Authors:** Fraser J. Graham, Pierpaolo Pellicori, Paul R. Kalra, Ian Ford, Dario Bruzzese, John G.F. Cleland

**Affiliations:** ^1^ Robertson Centre for Biostatistics University of Glasgow Glasgow UK; ^2^ School of Cardiovascular and Metabolic Health University of Glasgow Glasgow UK; ^3^ Department of Cardiology Portsmouth Hospitals University NHS Trust Portsmouth UK; ^4^ College of Medical, Veterinary & Life Sciences University of Glasgow Glasgow UK; ^5^ Faculty of Science and Health University of Portsmouth Portsmouth UK; ^6^ Department of Public Health University of Naples Federico II Naples Italy

**Keywords:** Iron deficiency, Intravenous iron, Heart failure, IRONMAN, Meta‐analysis

## Abstract

**Aims:**

For patients with heart failure (HF) and iron deficiency (ID), randomized trials suggest that intravenous (IV) iron reduces hospitalizations for heart failure (HHF), but uncertainty exists about the effects in subgroups and the impact on mortality. We conducted a meta‐analysis of randomized trials investigating the effect of IV iron on clinical outcomes in patients with HF.

**Methods and results:**

We identified randomized trials published between 1 January 2000 and 5 November 2022 investigating the effect of IV iron versus standard care/placebo in patients with HF and ID in any clinical setting, regardless of HF phenotype. Trials of oral iron or not in English were not included. The main outcomes of interest were a composite of HHF and cardiovascular death (CVD), on HHF alone and on cardiovascular and all‐cause mortality. Ten trials were identified with 3373 participants, of whom 1759 were assigned to IV iron. IV iron reduced the composite of recurrent HHF and CVD (rate ratio 0.75, 95% confidence interval [CI] 0.61–0.93; *p* < 0.01) and first HHF or CVD (odds ratio [OR] 0.72, 95% CI 0.53–0.99; *p* = 0.04). Effects on cardiovascular (OR 0.86, 95% CI 0.70–1.05; *p* = 0.14) and all‐cause mortality (OR 0.93, 95% CI 0.78–1.12; *p* = 0.47) were inconclusive. Results were similar in analyses confined to the first year of follow‐up, which was less disrupted by the COVID‐19 pandemic. Subgroup analyses found little evidence of heterogeneity for the effect on the primary endpoint, although patients with transferrin saturation <20% (OR 0.67, 95% CI 0.49–0.92) may have benefited more than those with values ≥20% (OR 0.99, 95% CI 0.74–1.30) (heterogeneity *p* = 0.07).

**Conclusion:**

In patients with HF and ID, this meta‐analysis suggests that IV iron reduces the risk of HHF but whether this is associated with a reduction in cardiovascular or all‐cause mortality remains inconclusive.

## Graphical abstract

In a meta‐analysis of ten randomized controlled trials (RCTs) including AFFIRM‐AHF and IRONMAN of over 3000 patients with heart failure (HF) and iron deficiency (ID), compared to standard care/placebo, intravenous (IV) iron reduced the primary outcome of recurrent hospitalisations for heart failure (HHF) and cardiovascular mortality (CVM) by 25%. The effect was mainly driven by a reduction in HHF with the effect on CVM being inconconclusive. Created in BioRender.com. Additional icons provided from http://icon‐library.com/icon/heart‐disease‐icon‐3.html.html Heart Disease Icon #293840. CI, confidence interval; CVM, cardiovascular mortality; HF, heart failure; HHF, hospitalization for heart failure; I, iron deficiency; IV, intravenous/interval variable; OR, odds ratio; RCT, randomized controlled trial; RR, risk rate; SE, standard error; SoC, standard of care.

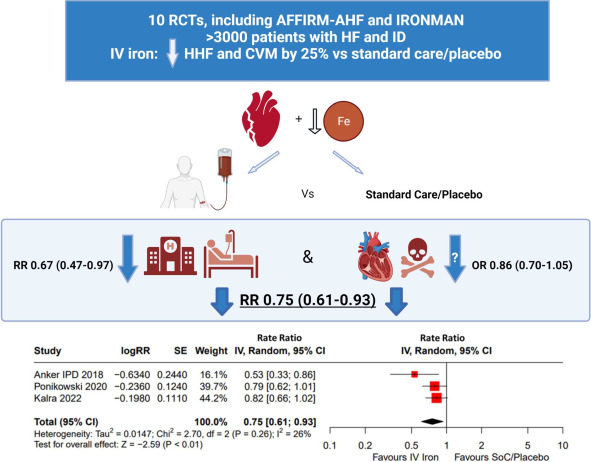

## Introduction

Amongst patients with heart failure, iron deficiency is common, ranging from 44% to 75% depending on the definition applied, severity of heart failure and left ventricular phenotype.^1^ Iron deficiency is associated with worse symptoms, poorer quality of life and impaired exercise capacity, which consistently improved with the administration of intravenous (IV) iron in randomized trials of up to 12‐months duration.^2^, ^3^, ^4^ Iron deficiency, irrespective of the presence of anaemia, is also associated with higher mortality in patients with heart failure.^5^ Current European^6^ and American^7^ guidelines recommend administration of IV iron to symptomatic patients with a reduced left ventricular ejection fraction and iron deficiency to improve symptoms, exercise capacity and quality of life.

Two recently published trials, each of which enrolled more than 1000 patients with heart failure and reduced or mildly reduced ejection fraction, investigated the effects of IV iron on morbidity and mortality.^8^, ^9^ Both trials narrowly failed to reach their primary endpoint; recurrent hospitalization for heart failure (HHF) or cardiovascular death. However, the conduct of each trial was disrupted by the COVID‐19 pandemic; pre‐specified sensitivity analyses found a nominally significant reduction in the primary endpoint in both cases.

In view of the inconclusive effects of IV iron on morbidity and mortality for patients with heart failure and iron deficiency, overall and in key patient subgroups, we conducted an updated systematic review and meta‐analysis, using aggregate data from peer‐reviewed published trials.

## Methods

The study protocol was registered prior to data extraction and analysis in PROSPERO (registration number: CRD42022358299).

Using pre‐specified search terms (online supplementary *Appendix* *S1*), we searched Medline for randomized trials written in English and published in peer‐reviewed journals between 1 January 2000 and 5 November 2022. The inclusion criteria for this meta‐analysis were informed by, and are identical to, a previously published meta‐analysis.^10^ In short, we included only randomized trials investigating the effects of IV iron versus either standard of care or placebo in patients with heart failure and iron deficiency, irrespective either of the definition of iron deficiency, participants' left ventricular ejection fraction and the formulation of IV iron.

Trials investigating the use of oral iron in patients with heart failure and iron deficiency were excluded as oral iron will not rapidly and reliably correct iron deficiency, is not included in international guidelines for treatment of iron deficiency in heart failure and would introduce further trial heterogeneity that could complicate interpretation of results.

In some smaller trials of IV iron, mortality was not specifically reported but serious adverse events, including HHF were; if this was the case, it was assumed that no deaths had occurred in these trials. When the cause of death was not clearly reported, it was adjudicated independently by two authors (FJG and PP), both of whom are experienced in clinical endpoint adjudication, based on the clinical information provided by authors in the text.

Two reviewers (FJG and PP) independently extracted the data according to the pre‐specified search terms. If there was failure of consensus, the opinion of a third independent reviewer was sought (JGFC or PRK). Data extracted included the number of patients and events in each trial arm and/or hazard ratios (HR) and 95% confidence intervals (CI) or rate ratios (RR) and incidence per 100 patient‐years at risk.

A composite of recurrent HHF and cardiovascular mortality, the primary endpoint of the two largest trials, was the primary outcome of this analysis. Secondary outcomes included a composite of HHF or cardiovascular mortality as a first event only, recurrent HHF only, cardiovascular mortality and all‐cause death. Because follow‐up was greater than 1 year only in IRONMAN, we also conducted analysis restricted to a follow‐up of 1 year or less. Published data from an individual patient data meta‐analysis of four trials^4^ was used for some recurrent events analyses when these outcomes were not found in the original trial reports or ClinicalTrials.gov. Provided information was available and thresholds were generally concordant, the effect of IV iron on the primary outcome in pre‐specified subgroups was analysed using data from IRONMAN, AFFIRM‐AHF and the individual patient data meta‐analysis noted above.^4^

A risk of bias assessment was conducted by two independent reviewers (FJG and PP) for the composite outcome of first HHF or cardiovascular death. Discrepancies were resolved by discussion and/or involving a third reviewer. Statistical analysis was conducted with RevMan Version 5.0 (Cochrane Collaboration, 2020) and R version 4.1.3. Both random and fixed effects analysis were used. In random effects models, the Paule–Mandel method to estimate variance heterogeneity was used. For recurrent events analyses, RR and corresponding standard errors were calculated from available RR and 95% CI in tables or text of reporting trials and analysed using inverse variance weighting. Results are displayed as RR and 95% CI or odd ratios (OR) and 95% CI for dichotomous outcomes. Heterogeneity was assessed by *I* ^2^ analysis.

## Results

Ten trials fulfilling the inclusion criteria were identified that provided data on 3373 participants (IV iron: *n* = 1759; standard of care or placebo: *n* = 1614) (online supplementary *Figure* *S1*).^2^, ^3^, ^8^, ^9^, ^11^, ^12^, ^13^, ^14^, ^15^, ^16^ Length of follow‐up ranged from 12 to 52 weeks with a median length of follow‐up in IRONMAN of 140 weeks (*Table* *1*). The most common definition of iron deficiency applied was a serum ferritin <100 μg/L regardless of transferrin saturation (TSAT) or a TSAT <20% if ferritin was 100–300 μg/L. IRONMAN had similar inclusion criteria but allowed inclusion of patients with a TSAT <20%, provided ferritin was <400 μg/L. Four trials compared ferric carboxymaltose (FCM) to placebo, three compared FCM to standard care, one trial compared ferric derisomaltose (FDI) to standard care, one trial compared iron sucrose to placebo and another to standard care. Trials generally excluded patients with severe anaemia (haemoglobin <9 g/dl), because their clinical need for correction of iron deficiency was considered too great, and those with a haemoglobin >14–15 g/dl, in whom iron deficiency appears uncommon.

**Table 1:** Characteristics of included trials

	Toblli et al.^16^	FERRIC‐HF^15^	FAIR‐HF^2^	CONFIRM‐HF^3^	EFFECT‐HF^12^	PRACTICE‐ASIA‐HF^13^	AFFIRM‐AHF^8^	Dhoot et al.^14^	IRON‐CRT^11^	IRONMAN^9^
Year of publication	2007	2008	2009	2015	2017	2018	2020	2020	2021	2022
Country	Argentina	UK and Poland	Europe and Argentina	Europe	Europe and Australia	Singapore	15 countries (international)	India	Belgium	UK
No. of patients (IV iron: control)	40 (1:1)	35 (2:1)	459 (2:1)	301 (1:1)	174 (1:1)	49 (1:1)	1108 (1:1)	70 (1:1)	75 (1:1)	1137 (1:1)
Double‐blind	Yes	No	Yes	Yes	No	No	Yes	No	Yes	No
Definition of ID	F <100 and/or T ≤20%	F <100 or T <20% + F 100–300	F <100 or T <20% + F 100–299	F <100 or T <20% + F 100–300	F < 100 or T <20% + F 100–300	T <20% and F <300	F <100 or T <20% + F 100–299	F <100 or T <20% + F 100–299	F <100 or T <20% + F 100–300	T <20% + F <400 or F <100
Main inclusion criteria (Hb: g/dl)	LVEF ≤35% NYHA II–IV Anaemia	LVEF ≤45% NYHA II–III Hb ≤14.5	LVEF ≤45% NYHA II–III Hb 9.5–13.5	LVEF ≤45% NYHA II/III Hb <15	LVEF ≤45% NYHA II or III Hb <15	HHF Hb <14	LVEF <50% HHF ↑NT‐proBNP Hb 8–15	NYHA II or III Age 18–65 Hb >8	CRT >6/12 LVEF <45% NYHA ≥II	LVEF ≤45% HHF ≤6/12 or ↑NT‐proBNP Hb 9–14
Age (years)	75	63	68	70	64	63	71	53	73	73
Women (%)	–	29	54	47	25	22	45	42	34	26
Ischaemic aetiology (%)	63	74	80	83	–	–	47	31	57	57
LVEF (%)	31 ± 4	30 ± 7	32 ± 6	37 ± 8	33 ± 9	39 ± 18	33 (10)	24.9 ± 5	33 ± 8	32 (25–37)^a^
NT‐proBNP (pg/ml)	256 ± 125	–	–	2511 ± 5006	1576^a^	–	4743 (2781–8128)^a^	–	2227 (299–2967)	–
eGFR (ml/min/1.73 m^2^)	–	–	64	66	52	–	–	–	56 ± 25	52 (38–86)^a^
Hb (g/dl)	10.3 ± 0.6	12.6 ± 1.2	11.9 ± 1.3	12.3 ± 1.4	12.9 ± 1.3	11.6 ± 1.9	12.3 ± 1.6	11 ± 4	13.3 ± 1.2	12.1 (11.2–12.8)^a^
Ferritin (μg/L)	73 ± 30	62 ± 37	53 ± 55	57 ± 48	48^a^	91 ± 80	84 ± 62	40 ± 27	82^a^	49 (30–86)^a^
TSAT (%)	20 ± 1	20 ± 8	18 ± 13	20 ± 18	17^a^	16 ± 10	15 ± 8	–	18.8 ± 6	15 (11–20)^a^
Form of iron therapy (mean dose)	Iron sucrose; 1000 mg	Iron sucrose; 1433 mg	FCM; N/A	FCM; 1500 mg	FCM; 1204 mg	FCM; 1000 mg	FCM; 1352 mg	FCM; N/A	FCM; 959 mg	FDI; 1978 mg^b^
Follow‐up (weeks)	24	18	24	52	24	12	52	12	12	140 (94–187)^a^
Outcomes reported										
HHF	+	+	+	+	+	+	+	+	+	+
CVM	−	+	+	+	+	−	+	+	+	+

CVM, cardiovascular mortality; eGFR, estimated glomerular filtration rate; F, ferritin; FCM, ferric carboxymaltose; FDI, ferric derisomaltose; Hb, haemoglobin; HHF, hospitalization for heart failure; ID, iron deficiency; IV, intravenous; LVEF, left ventricular ejection fraction; N/A, not available; NT‐proBNP, N‐terminal pro‐B‐type natriuretic peptide; NYHA, New York Heart Association; T/TSAT, transferrin saturation.

If laboratory values were not available for whole study populations, values from the intervention group were reported.

^a^ Median and (Q1–Q3) reported.

^b^ Mean dose in Year 1.

### Hospitalizations for heart failure or cardiovascular mortality

**Figure 1:**
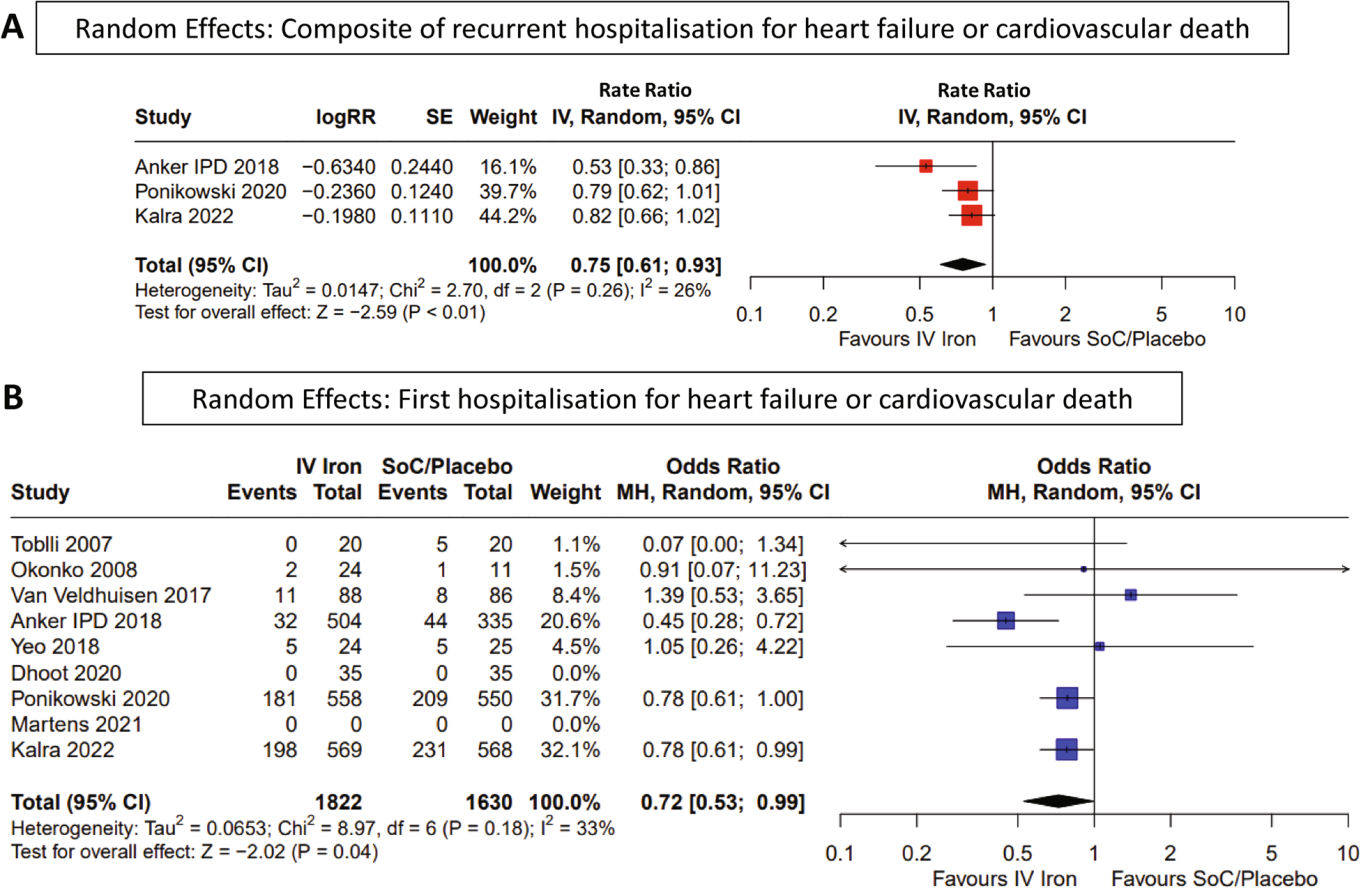
Random effects: (*A*) composite of recurrent hospitalization for heart failure or cardiovascular death, and (*B*) first hospitalization for heart failure or cardiovascular death. CI, confidence interval; IPD, individual patient data; IV, intravenous/interval variable; MH, Mantel–Haenszel; RR, rate ratio; SE, standard error; SoC, standard of care.

**Figure 2:**
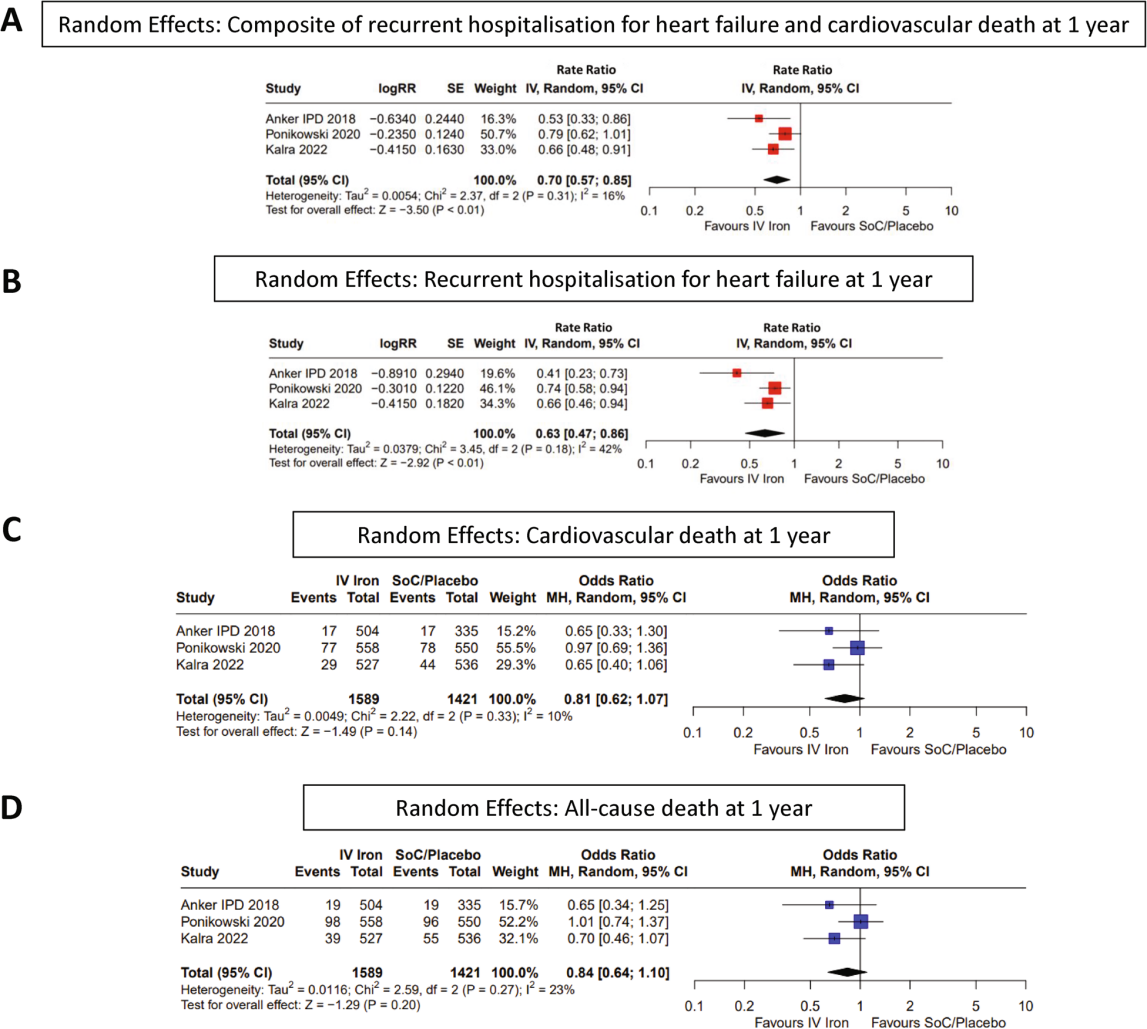
Random effects: (*A*) composite of recurrent hospitalization for heart failure and cardiovascular death at 1 year, (*B*) recurrent hospitalization for heart failure at 1 year, (*C*) cardiovascular death at 1 year, and (*D*) all‐cause death at 1 year. CI, confidence interval; IPD, individual patient data; IV, intravenous/interval variable; MH, Mantel–Haenszel; RR, rate ratio; SE, standard error; SoC, standard of care.

Analysis of the primary outcome included 1631 patients assigned to IV iron and 1453 assigned to the control group from the AFFIRM‐AHF and IRONMAN trials and the individual patient data meta‐analysis. IV iron reduced the composite of recurrent HHF or cardiovascular death (RR 0.75 [0.61–0.93], *p* < 0.01) (*Figure* *1* and *Graphical Abstract*). Repeating the analysis in fixed effects models did not substantially alter the result (online supplementary *Figure* *S2*). Results of the recurrent event analysis were similar when restricted to 1 year (RR 0.70 [0.57–0.85], *p* < 0.01) (*Figure* *2*). Overall, the primary composite occurred in 429 (24%) patients randomized to IV iron and 503 (31%) randomized to the control group (OR 0.72 [0.53–0.99], *p* = 0.04). There was no evidence of heterogeneity according to preparation of iron used, with the largest number of patients receiving either FCM or FDI.

### Recurrent hospitalizations for heart failure alone

**Figure 3:**
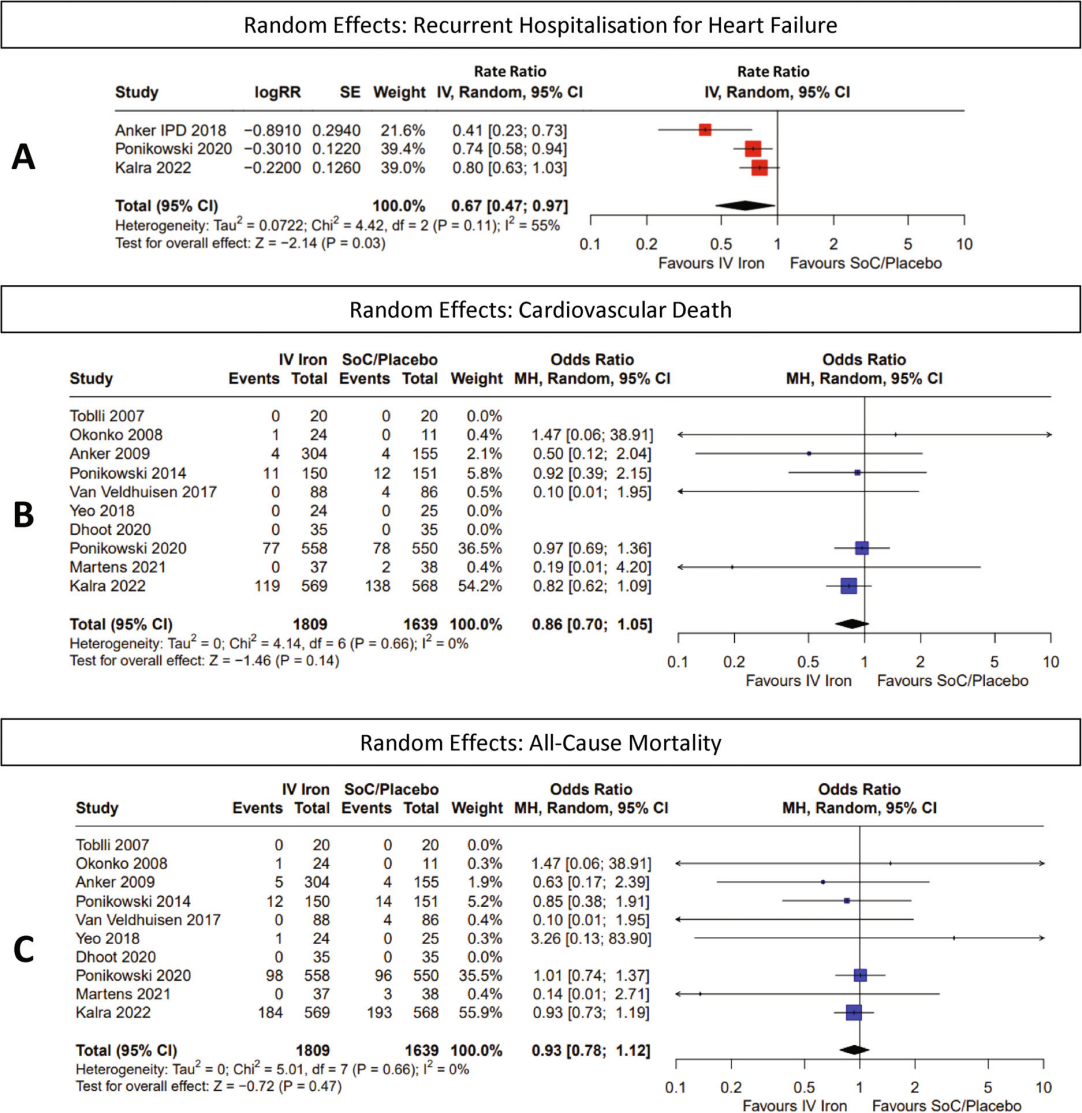
Random effects: (*A*) recurrent hospitalization for heart failure, (*B*) cardiovascular death, and (*C*) all‐cause mortality. CI, confidence interval; IPD, individual patient data; IV, intravenous/interval variable; MH, Mantel–Haenszel; RR, rate ratio; SE, standard error; SoC, standard of care.

Recurrent HHF overall (RR 0.67 [0.47–0.97], *p* = 0.03) (*Figure* *3*) and restricted to the first year of follow‐up (RR 0.63 [0.47–0.86], *p* < 0.01) (*Figure* *2*) were reduced substantially. Results were similar in fixed effects models (online supplementary *Figures* *S3* and *S4*).

### Cardiovascular and all‐cause mortality

The effects of IV iron on cardiovascular death overall (OR 0.86 [0.70–1.05], *p* = 0.14) or at 1 year (OR 0.81 [0.62–1.07], *p* = 0.14) or on all‐cause death overall (OR 0.93 [0.78–1.12], *p* = 0.47) or at 1 year (OR 0.84 [0.64–1.10], *p* = 0.20) were inconclusive and do not exclude substantial reductions or some excess risk (*Figures* *2* and *3*). Repeating analysis in fixed effects models did not materially alter results (online supplementary *Figures* *S3* and *S4*).

### Subgroup analysis

**Figure 4:**
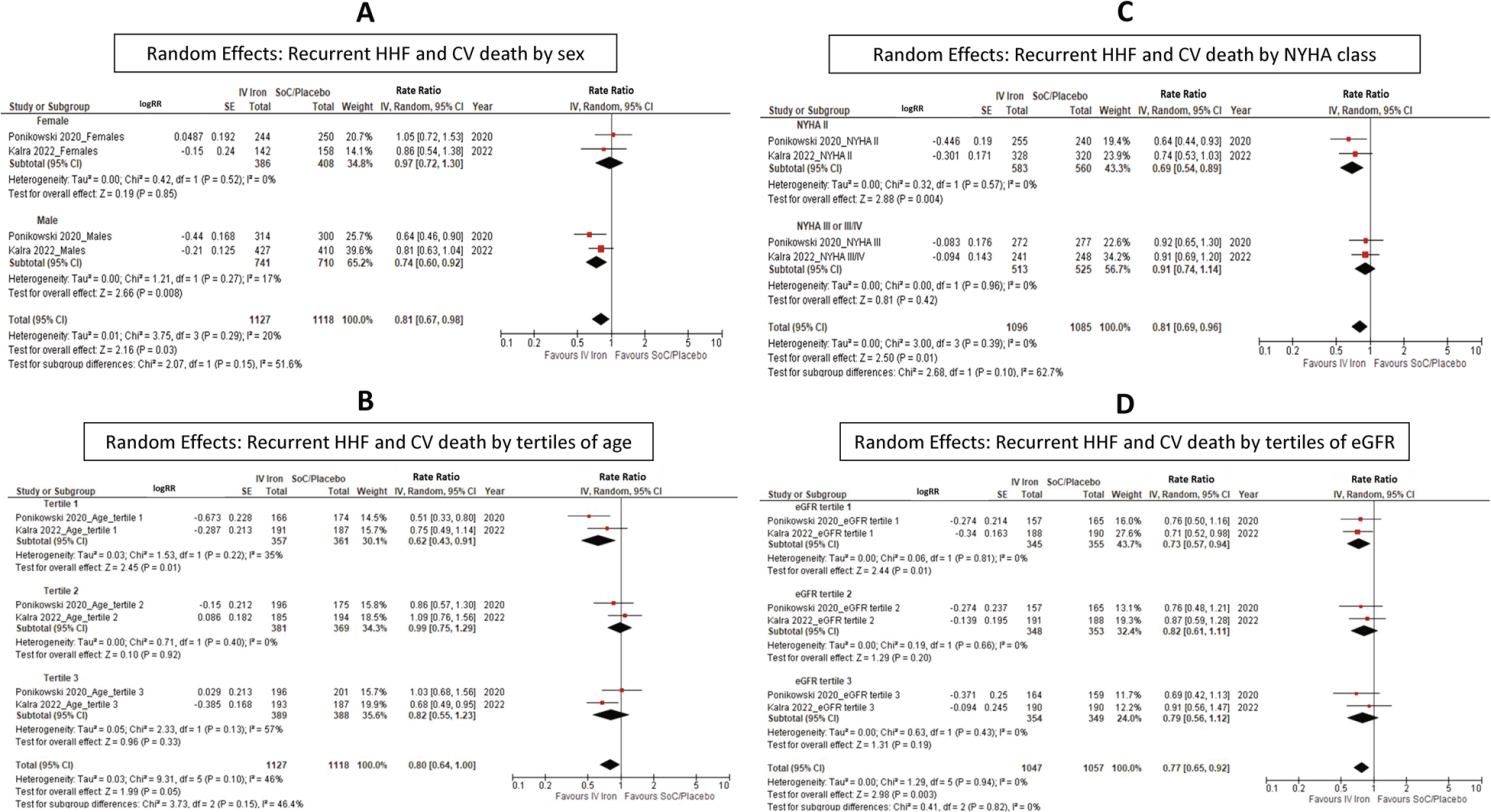
Random effects: (*A*) recurrent hospitalization for heart failure (HHF) and cardiovascular (CV) death by sex, (*B*) recurrent HHF and CV death by tertiles of age, (*C*) recurrent HHF and CV death by New York Heart Association (NYHA) class, and (*D*) recurrent HHF and CV death by tertiles of estimated glomerular filtration rate (eGFR). CI, confidence interval; IV, intravenous/interval variable; RR, rate ratio; SE, standard error; SoC, standard of care.

**Figure 5:**
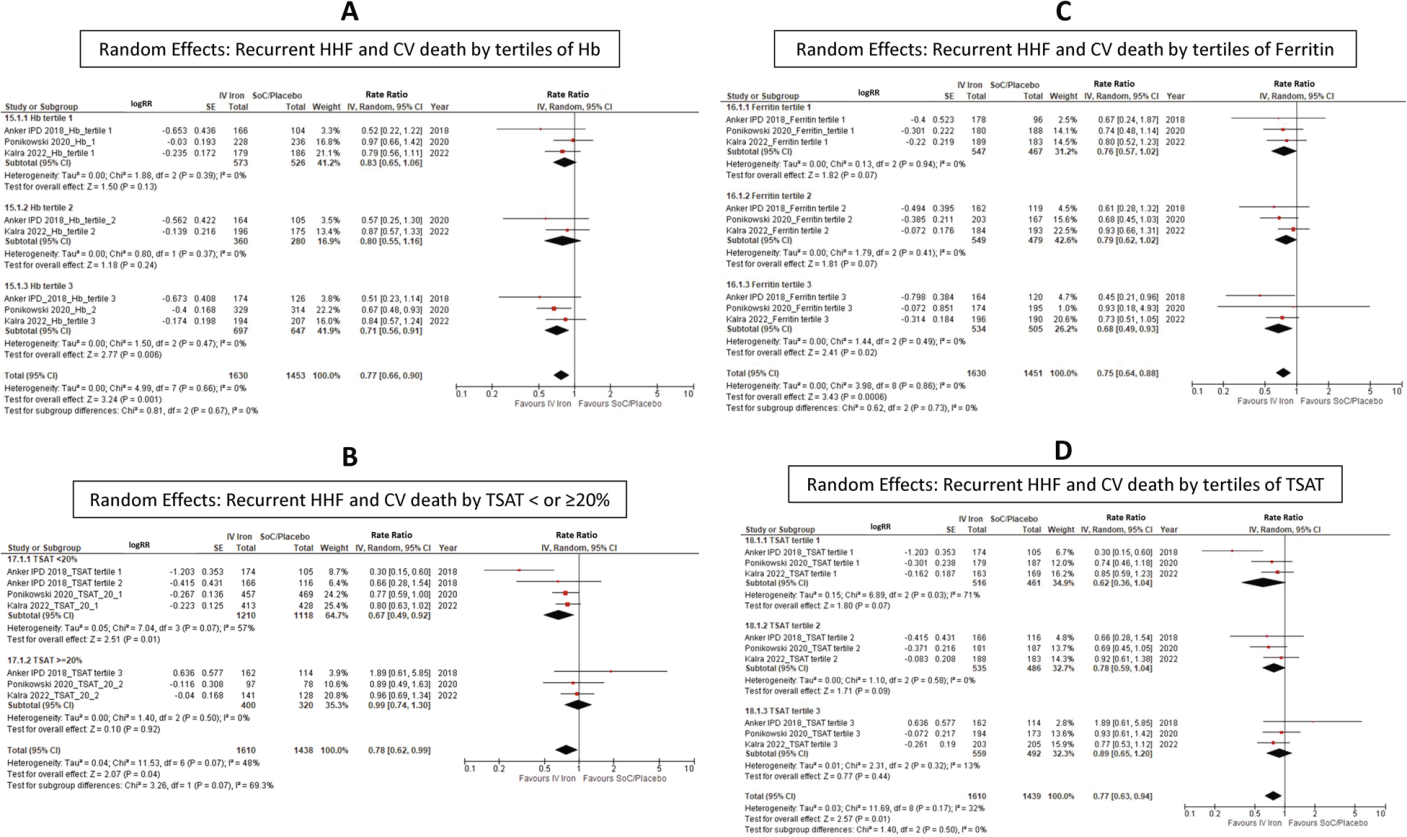
Random effects: (*A*) recurrent hospitalization for heart failure (HHF) and cardiovascular (CV) death by tertiles of haemoglobin (Hb), (*B*) recurrent HHF and CV death by transferrin saturation (TSAT) <20% or ≥20%, (*C*) recurrent HHF and CV death by tertiles of ferritin, and (*D*) recurrent HHF and CV death by tertiles of TSAT. CI, confidence interval; IPD, individual patient data; IV, intravenous/interval variable; RR, rate ratio; SE, standard error; SoC, standard of care.

**Figure 6:**
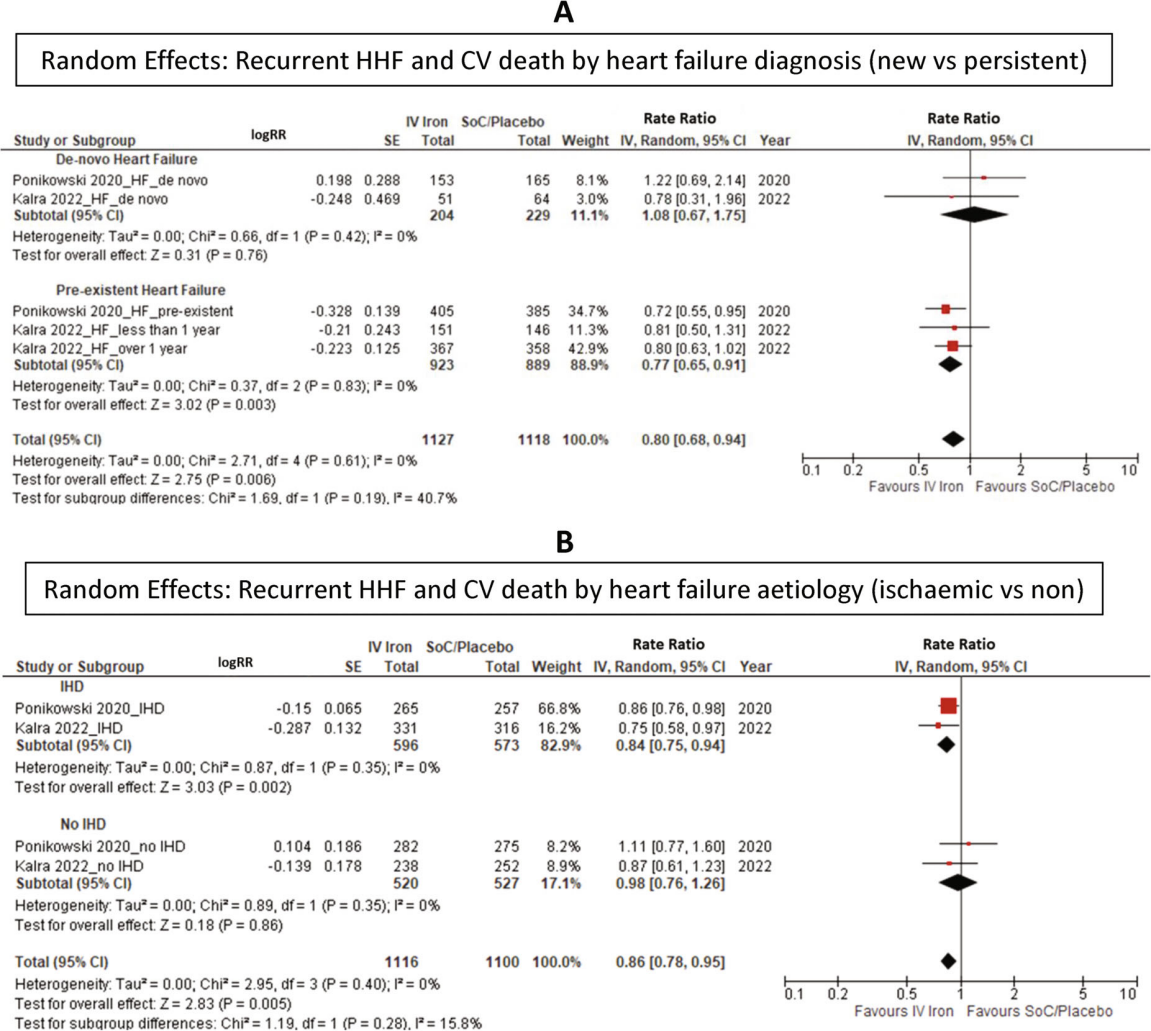
Random effects: (*A*) recurrent hospitalization for heart failure (HHF) and cardiovascular (CV) death by heart failure diagnosis (new vs. persistent), (*B*) recurrent HHF and CV death by heart failure aetiology (ischaemic vs. non‐ischaemic). CI, confidence interval; HF, heart failure; IHD, ischaemic heart disease; IV, intravenous/interval variable; RR, rate ratio; SE, standard error; SoC, standard of care.

No statistically significant difference in effect amongst subgroups was identified (*Figures* *4*, *5*, *6*). There was a trend to more substantial benefit from IV iron in those with ischaemic heart disease (OR 0.84 [0.75–0.94]) compared to those with other aetiologies of heart failure (OR 0.98 [0.76–1.26]) but this did not achieve statistical significance (*p* = 0.28). Patients with a low TSAT (<20%) (OR 0.67 [0.49–0.92]) also appeared to benefit more from IV iron versus those with a TSAT ≥20% (OR 0.99 [0.74–1.30]) but, again, this did not achieve statistical significance (*p* = 0.07). Results obtained from fixed effects models were similar (online supplementary *Figures* *S5–S7*).

Risk of bias assessment identified three trials to be of some concern (online supplementary *Figure* *S8*). Combined events from these trials comprised <2% of the total in the composite secondary analysis.

## Discussion

This meta‐analysis suggests that IV iron reduces the risk of HHF substantially. However, the effect of IV iron on cardiovascular or all‐cause mortality is inconclusive, with the 95% CI around the point‐estimate unable to exclude a substantial reduction or some excess risk. We did not observe marked heterogeneity in the effect of IV iron on the composite of HHF or cardiovascular mortality in pre‐specified subgroups, although patients with a low TSAT might have obtained greater benefit.

The benefits of IV iron were observed most clearly in the first year after randomization. There are many potential explanations why the effect of IV iron might have waned over time, the most important being the disruption caused by COVID‐19 that prevented many patients from receiving repeated doses of iron to maintain repletion. Oral iron supplements and non‐protocol administration of IV iron to control groups may also have attenuated the effect observed in trials. Iron deficiency may resolve in response to effective treatment for congestion and heart failure.^17^ It is also possible that those most able to respond to IV iron do so in the first year with a shrinking pool of responders thereafter.

Inconsistencies exist between international guidelines with respect to treatment with intravenous iron. For example, the 2022 AHA/ACC/HFSA guidelines recommend intravenous iron to improve functional status and quality of life (strength 2a) but do not mention effects on HHF or endorse any particular preparation.^7^ In contrast, the 2021 European Society for Cardiology guideline recommended FCM as the only intravenous iron preparation that should be considered to improve symptoms and exercise capacity for patients with heart failure and left ventricular ejection fraction ≤45% (Class IIa, Level A).^6^ In addition, following publication of the AFFIFRM‐AHF trial,^8^ the European Society for Cardiology guideline states that FCM should be considered to reduce the risk of further hospital admissions for heart failure in patients recently hospitalized due to heart failure (Class IIa, Level B).^6^ The data now show a meaningful and consistent reduction in risk of heart failure admission with at least two iron preparations, in a broad range of patients with heart failure and reduced or mildly reduced ejection fraction, including outpatients without recent hospitalization.

The precise criteria that best reflect iron deficiency and therefore identify patients who have the most to gain from IV iron are uncertain.^1^, ^17^, ^18^, ^19^ An individual patient data meta‐analysis of smaller, short‐term trials suggested that a TSAT <20% might better identify patients who benefit from IV iron than do the current, predominantly ferritin‐based, definitions used to include patients in randomized trials of heart failure.^4^ This meta‐analysis provides further, albeit inconclusive, support for using TSAT to select patients most likely to benefit from IV iron. Unfortunately, none of the trials have yet reported the effects of treatment according to serum iron, which is a strong marker of prognosis in the general population^20^ and in patients with heart failure.^1^, ^17^, ^18^, ^19^

More than 40 years ago, it was suggested that iron deficiency might account for the lower risk of ischaemic heart disease in women.^21^, ^22^ However, subsequent research found that iron deficiency might increase the risk of developing ischaemic heart disease.^23^, ^24^ The AFFIRM‐AHF trial suggested that patients with ischaemic heart disease might benefit more from IV iron, although this might simply reflect the higher risk and events rates of patients with heart failure due to ischaemic heart disease or its association with more severe iron deficiency.^25^ Although a trend to greater benefit with IV iron for patients with ischaemic heart disease was also observed in the IRONMAN trial, our meta‐analysis failed to demonstrate significant heterogeneity according to the cause of left ventricular dysfunction.

More trials of IV iron in patients with heart failure and a reduced (NCT03037931; NCT03036462) or preserved (NCT03074591) left ventricular ejection fraction will report in the next few years, which may help confirm or refute an effect of IV iron on cardiovascular or all‐cause mortality, overall or in specific subgroups of patients, such as those with a TSAT <20% or with ischaemic heart disease.

### Limitations

Some events were identified only from secondary reports of trial. The effect of IV iron on cardiovascular hospitalizations was not assessed as it was defined differently in AFFIRM‐AHF and IRONMAN. The effect of IV iron on first HHF was only assessed at 1 year, because this outcome was not reported at full follow‐up in IRONMAN. This was a meta‐analysis of aggregate data for each trial, rather than individual patient data, and therefore cannot investigate interactions amongst variables. Results obtained from analyses including data derived from the individual patient data meta‐analysis by Anker *et al*.^4^ may restrict the estimation of between‐trial heterogeneity. This should be kept in mind when interpreting these results. Thresholds for tertiles from individual trials were not identical when conducting subgroup analyses. Also, any differences observed in subgroup analyses should be considered hypothesis‐generating because of the limited amount of data.

## Conclusion

In aggregate data of patients with heart failure from 10 randomized trials, IV iron reduced the risk of the composite of both first and recurrent HHF or cardiovascular death, a result that is driven primarily by a reduction in HHF.

### Funding

P.P. and J.G.F.C. are supported by the British Heart Foundation Centre of Research Excellence (RE/18/6134217). F.J.G., P.P. and J.G.F.C. have been awarded a project grant from the British Heart Foundation to assess the prevalence of iron deficiency in patients undergoing elective cardiac surgery (PG/2019/35089).

**Conflict of interest**: F.J.G. reports receipt of sponsorship from Pharmacosmos to attend an international meeting. P.P. has received a research grant (Scotland Grant) from Heart Research UK; he reports consulting fees from Vifor and Pharmacosmos. P.R.K. reports research grants from British Heart Foundation and Pharmacosmos; consulting fees from Ackea, Amgen, Boehringer Ingelheim, Pharmacosmos, Servier, and Vifor Pharma; payment for lectures from AstraZeneca, Bayer, Novartis, Pfizer, Pharmacosmos and Vifor Pharma; support for attending meetings from Pharmacosmos. I.F. reports research grants from the BHF, Vifor and Pharmacosmos. D.B. has nothing to disclose. J.G.F.C. reports personal honoraria for lectures and advisory boards from Pharmacosmos, Vifor, from AstraZeneca, Amgen, Bayer, Novartis and Servier.

## Supporting information


**Appendix S1.** Supplementary Information.
