# Supplementary material for: Intravenous iron in patients with heart failure and iron deficiency: an updated meta‐analysis
Source: Eur J Heart Fail. 2023 Mar 8;25(4):528–37. doi: 10.1002/ejhf.2810 (PMC10946839; doi:10.1002/ejhf.2810)
Supplement: Supplementary file 1 — Appendix S1. Supplementary Information. [file EJHF-25-528-s001.pdf]

Pre-specified search terms: (((((((((heart failure)) AND (iron deficiency)) OR (iron repletion)) OR (intravenous iron)) OR (ferric carboxymaltose)) OR (ferric derisomaltose)) OR (iron isomaltoside 1000)) OR (iron sucrose)) OR (iron supplementation)) OR (iron therapy)

Filters: English Language; Clinical trial; Randomized trial, Humans, Medline

Date range: 1st January 2000 – 5th November 2022

Supplementary Figure S1

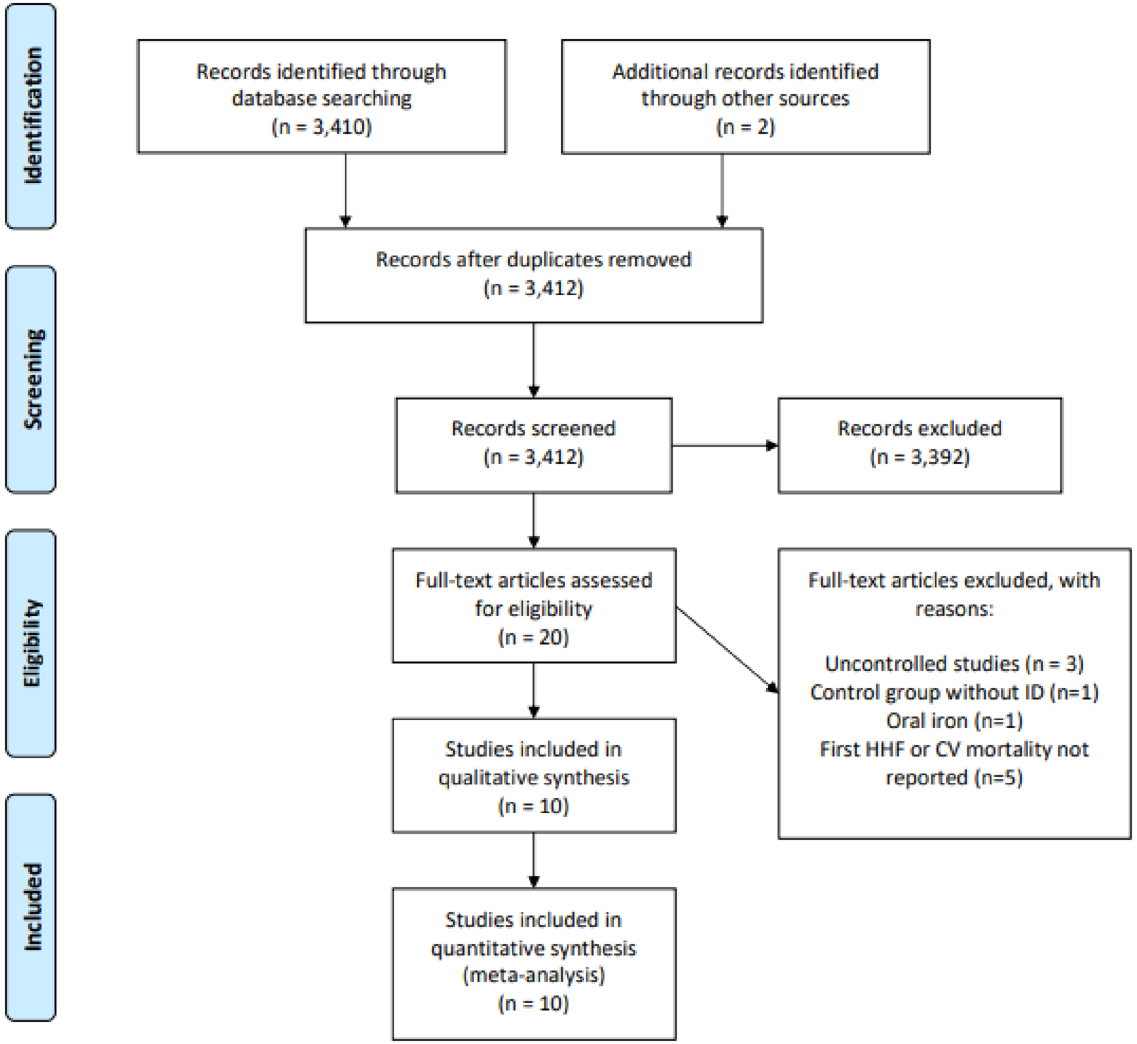

Modified from: Moher D, Liberati A, Tetzlaff J, Altman DG, The PRISMA Group (2009). *Preferred Reporting Items Systematic Reviews and Meta-Analyses*: The PRISMA Statement. PLoS Med 6(7): e1000097. doi:10.1371/journal.pmed1000097

Supplementary Figure S2

A

Fixed Effects: Composite of recurrent hospitalisation for heart failure and cardiovascular death

Study or Subgroup	logRR	SE	IV Iron Total	SoC/Placebo Total	Weight	Rate Ratio IV, Fixed, 95% CI	Year
Anker IPD 2018	-0.634	0.244	504	335	10.3%	0.53 [0.33, 0.86]	2018
Ponikowski 2020	-0.236	0.124	558	550	39.9%	0.79 [0.62, 1.01]	2020
Kalra 2022	-0.198	0.111	569	568	49.8%	0.82 [0.66, 1.02]	2022
**Total (95% CI)**			**1631**	**1453**	**100.0%**	**0.77 [0.66, 0.90]**	

Heterogeneity: Chi² = 2.70, df = 2 (P = 0.26); I² = 26%

Test for overall effect: Z = 3.30 (P = 0.0010)

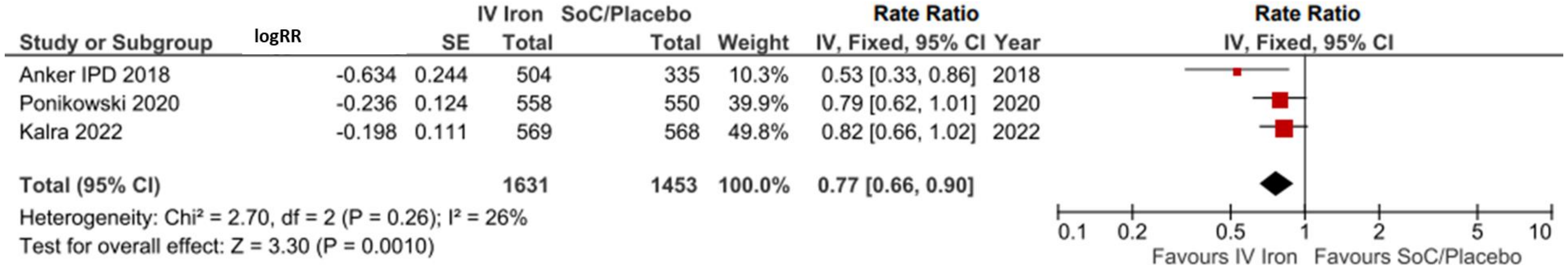

B

Fixed Effects: First hospitalisation for heart failure or cardiovascular death

Study or Subgroup	IV Iron Events	IV Iron Total	SoC/Placebo Events	SoC/Placebo Total	Weight	Odds Ratio M-H, Fixed, 95% CI	Year
Toblli 2007	0	20	5	20	1.5%	0.07 [0.00, 1.34]	2007
Okonko 2008	2	24	1	11	0.3%	0.91 [0.07, 11.23]	2008
Van Veldhuisen 2017	11	88	8	86	2.0%	1.39 [0.53, 3.65]	2017
Anker IPD 2018	32	504	44	335	13.7%	0.45 [0.28, 0.72]	2018
Yeo 2018	5	24	5	25	1.1%	1.05 [0.26, 4.22]	2018
Dhoot 2020	0	35	0	35		Not estimable	2020
Ponikowski 2020	181	558	209	550	39.5%	0.78 [0.61, 1.00]	2020
Martens 2021	0	0	0	0		Not estimable	2021
Kalra 2022	198	569	231	568	41.9%	0.78 [0.61, 0.99]	2022
**Total (95% CI)**		**1822**		**1630**	**100.0%**	**0.74 [0.63, 0.87]**	
Total events	429		503				

Heterogeneity: Chi² = 8.98, df = 6 (P = 0.17); I² = 33%

Test for overall effect: Z = 3.75 (P = 0.0002)

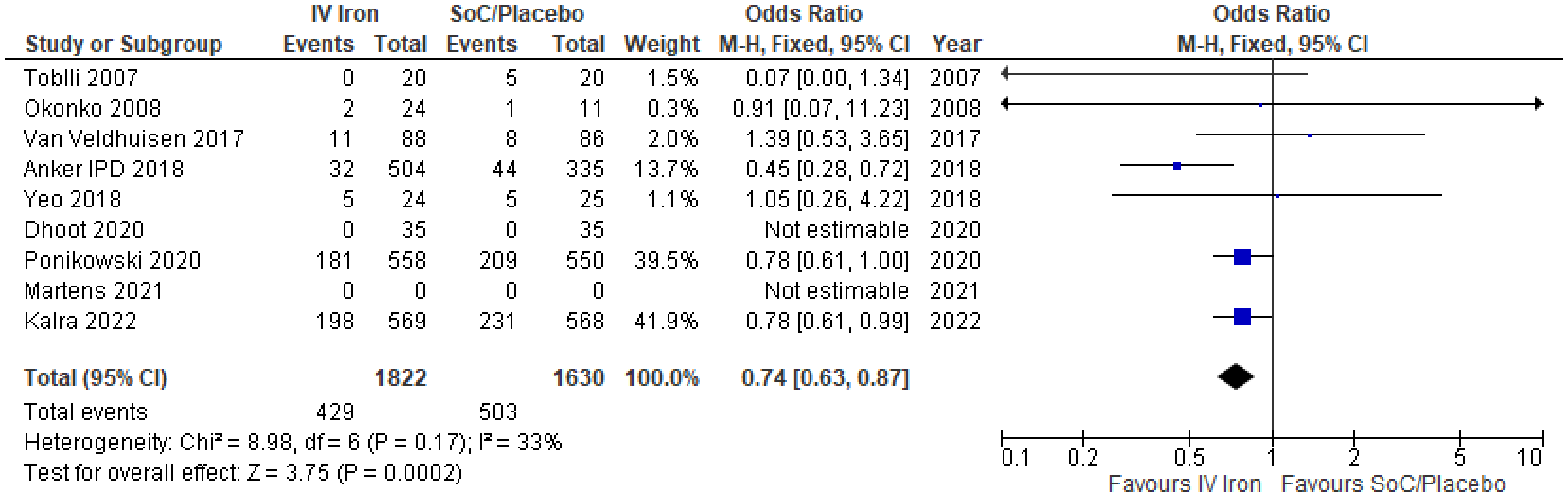

Supplementary Figure S3

A

Fixed Effects: Recurrent Hospitalisation for Heart Failure

Study or Subgroup	logRR	SE	IV Iron Total	SoC/Placebo Total	Weight	Rate Ratio IV, Fixed, 95% CI	Year
Anker IPD 2018	-0.891	0.294	504	335	8.2%	0.41 [0.23, 0.73]	2018
Ponikowski 2020	-0.301	0.122	558	550	47.4%	0.74 [0.58, 0.94]	2020
Kalra 2022	-0.22	0.126	569	568	44.4%	0.80 [0.63, 1.03]	2022
**Total (95% CI)**			**1631**	**1453**	**100.0%**	**0.73 [0.62, 0.86]**	

Heterogeneity: Chi² = 4.42, df = 2 (P = 0.11); I² = 55%
Test for overall effect: Z = 3.73 (P = 0.0002)

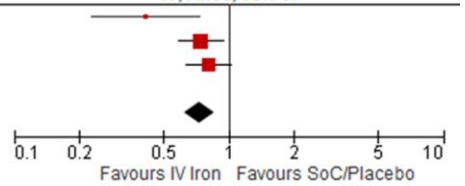

B

Fixed Effects: Cardiovascular Death

Study or Subgroup	IV Iron Events	IV Iron Total	SoC/Placebo Events	SoC/Placebo Total	Weight	Odds Ratio M-H, Fixed, 95% CI	Year
Toblli 2007	0	20	0	20		Not estimable	2007
Okonko 2008	1	24	0	11	0.3%	1.47 [0.06, 38.91]	2008
Anker 2009	4	304	4	155	2.6%	0.50 [0.12, 2.04]	2009
Ponikowski 2014	11	150	12	151	5.5%	0.92 [0.39, 2.15]	2014
Van Veldhuisen 2017	0	88	4	86	2.3%	0.10 [0.01, 1.95]	2017
Yeo 2018	0	24	0	25		Not estimable	2018
Dhoot 2020	0	35	0	35		Not estimable	2020
Ponikowski 2020	77	558	78	550	33.7%	0.97 [0.69, 1.36]	2020
Martens 2021	0	37	2	38	1.2%	0.19 [0.01, 4.20]	2021
Kalra 2022	119	569	138	568	54.4%	0.82 [0.62, 1.09]	2022
**Total (95% CI)**		**1809**		**1639**	**100.0%**	**0.85 [0.69, 1.04]**	
Total events	212		238				

Heterogeneity: Chi² = 4.16, df = 6 (P = 0.66); I² = 0%
Test for overall effect: Z = 1.59 (P = 0.11)

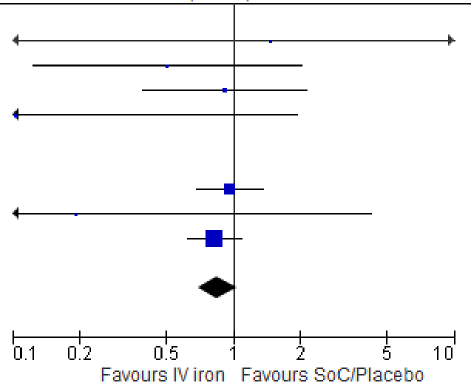

C

Fixed Effects: All-Cause Mortality

Study or Subgroup	IV Iron Events	IV Iron Total	SoC/Placebo Events	SoC/Placebo Total	Weight	Odds Ratio M-H, Fixed, 95% CI	Year
Toblli 2007	0	20	0	20		Not estimable	2007
Okonko 2008	1	24	0	11	0.3%	1.47 [0.06, 38.91]	2008
Anker 2009	5	304	4	155	2.2%	0.63 [0.17, 2.39]	2009
Ponikowski 2014	12	150	14	151	5.4%	0.85 [0.38, 1.91]	2014
Van Veldhuisen 2017	0	88	4	86	1.9%	0.10 [0.01, 1.95]	2017
Yeo 2018	1	24	0	25	0.2%	3.26 [0.13, 83.90]	2018
Dhoot 2020	0	35	0	35		Not estimable	2020
Ponikowski 2020	98	558	96	550	33.6%	1.01 [0.74, 1.37]	2020
Martens 2021	0	37	3	38	1.4%	0.14 [0.01, 2.71]	2021
Kalra 2022	184	569	193	568	55.0%	0.93 [0.73, 1.19]	2022
**Total (95% CI)**		**1809**		**1639**	**100.0%**	**0.92 [0.77, 1.11]**	
Total events	301		314				

Heterogeneity: Chi² = 5.02, df = 7 (P = 0.66); I² = 0%
Test for overall effect: Z = 0.85 (P = 0.39)

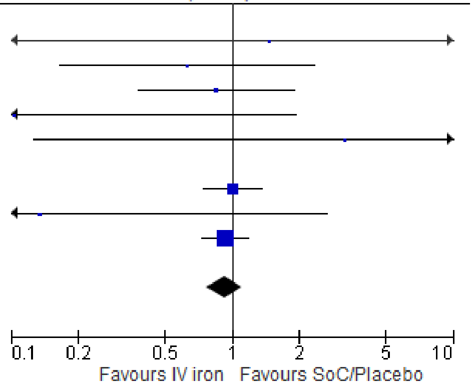

Supplementary Figure S4

Fixed Effects: Composite of recurrent hospitalisation for heart failure and cardiovascular death at 1 year

A

Study or Subgroup	logRR	SE	IV Iron Total	SoC/Placebo Total	Weight	Rate Ratio IV, Fixed, 95% CI	Year
Anker IPD 2018	-0.634	0.244	504	335	14.1%	0.53 [0.33, 0.86]	2018
Ponikowski 2020	-0.235	0.124	558	550	54.4%	0.79 [0.62, 1.01]	2020
Kalra 2022	-0.415	0.163	527	536	31.5%	0.66 [0.48, 0.91]	2022
**Total (95% CI)**			**1589**	**1421**	**100.0%**	**0.71 [0.59, 0.84]**	

Heterogeneity: Chi² = 2.37, df = 2 (P = 0.31); I² = 16%
Test for overall effect: Z = 3.80 (P = 0.0001)

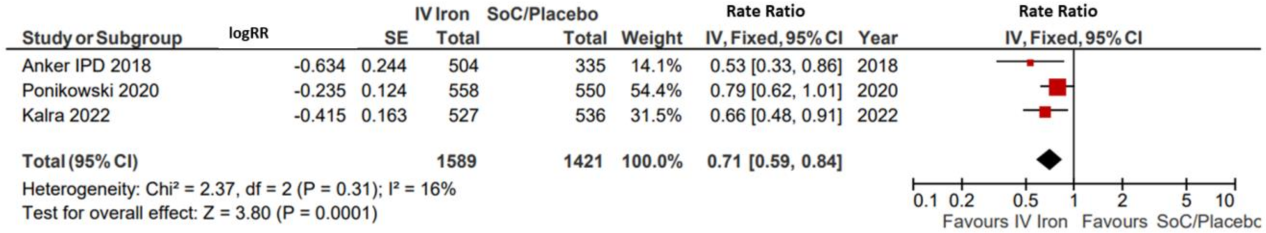

Fixed Effects: Recurrent hospitalisation for heart failure at 1 year

B

Study or Subgroup	logRR	SE	IV Iron Total	SoC/Placebo Total	Weight	Rate Ratio IV, Fixed, 95% CI	Year
Anker IPD 2018	-0.891	0.294	504	335	10.6%	0.41 [0.23, 0.73]	2018
Ponikowski 2020	-0.301	0.122	558	550	61.7%	0.74 [0.58, 0.94]	2020
Kalra 2022	-0.415	0.182	527	536	27.7%	0.66 [0.46, 0.94]	2022
**Total (95% CI)**			**1589**	**1421**	**100.0%**	**0.67 [0.56, 0.81]**	

Heterogeneity: Chi² = 3.45, df = 2 (P = 0.18); I² = 42%
Test for overall effect: Z = 4.13 (P < 0.0001)

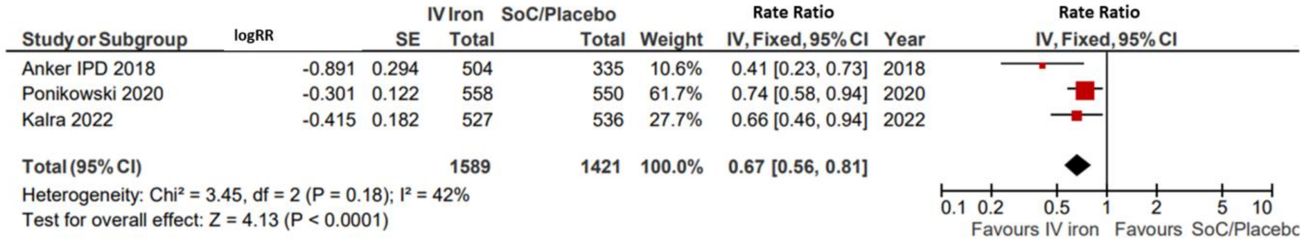

Fixed Effects: Cardiovascular death at 1 year

C

Study or Subgroup	IV Iron Events	IV Iron Total	SoC/Placebo Events	SoC/Placebo Total	Weight	Odds Ratio M-H, Fixed, 95% CI	Year
Anker IPD 2018	17	504	17	335	15.3%	0.65 [0.33, 1.30]	2018
Ponikowski 2020	77	558	78	550	52.6%	0.97 [0.69, 1.36]	2020
Kalra 2022	29	527	44	536	32.0%	0.65 [0.40, 1.06]	2022
**Total (95% CI)**		**1589**		**1421**	**100.0%**	**0.82 [0.63, 1.06]**	
Total events	123		139				

Heterogeneity: Chi² = 2.22, df = 2 (P = 0.33); I² = 10%
Test for overall effect: Z = 1.53 (P = 0.13)

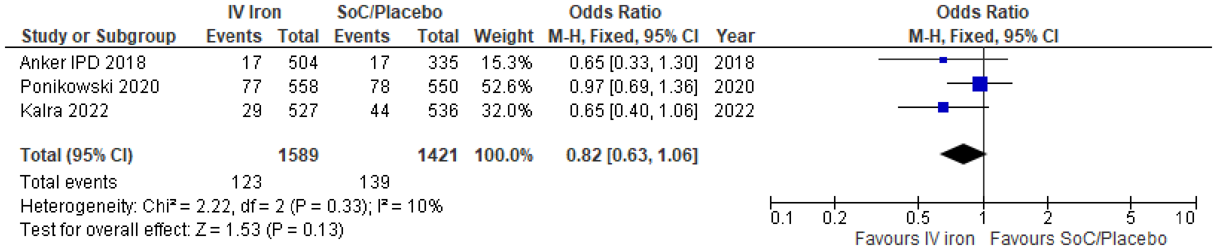

Fixed Effects: All-cause death at 1 year

D

Study or Subgroup	IV Iron Events	IV Iron Total	SoC/Placebo Events	SoC/Placebo Total	Weight	Odds Ratio M-H, Fixed, 95% CI	Year
Anker IPD 2018	19	504	19	335	14.4%	0.65 [0.34, 1.25]	2018
Ponikowski 2020	98	558	96	550	52.4%	1.01 [0.74, 1.37]	2020
Kalra 2022	39	527	55	536	33.2%	0.70 [0.46, 1.07]	2022
**Total (95% CI)**		**1589**		**1421**	**100.0%**	**0.85 [0.68, 1.08]**	
Total events	156		170				

Heterogeneity: Chi² = 2.59, df = 2 (P = 0.27); I² = 23%
Test for overall effect: Z = 1.33 (P = 0.18)

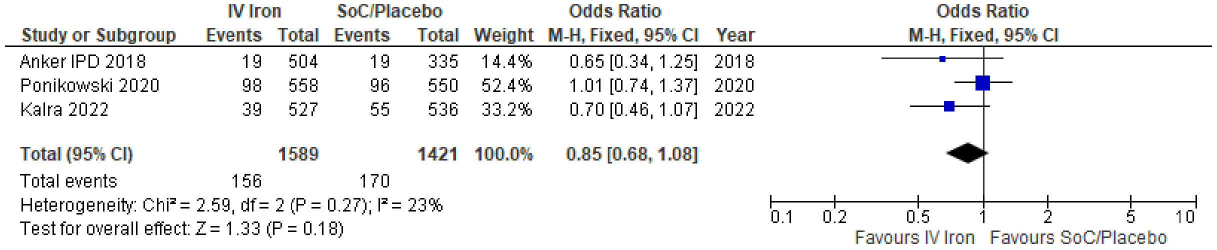

Supplementary Figure S5

A

Fixed Effects: Recurrent HHF and CV death by sex

Study or Subgroup	logRR	SE	IV Iron Total	SoC/Placebo Total	Weight	Rate Ratio IV, Fixed, 95% CI	Year
**Female**							
Ponikowski 2020_Females	0.0487	0.192	244	250	18.8%	1.05 [0.72, 1.53]	2020
Kalra 2022_Females	-0.15	0.24	142	158	12.1%	0.86 [0.54, 1.38]	2022
**Subtotal (95% CI)**			**386**	**408**	**30.9%**	**0.97 [0.72, 1.30]**	
Heterogeneity: Chi² = 0.42, df = 1 (P = 0.52); I² = 0%							
Test for overall effect: Z = 0.19 (P = 0.85)							
**Male**							
Ponikowski 2020_Males	-0.44	0.168	314	300	24.6%	0.64 [0.46, 0.90]	2020
Kalra 2022_Males	-0.21	0.125	427	410	44.5%	0.81 [0.63, 1.04]	2022
**Subtotal (95% CI)**			**741**	**710**	**69.1%**	**0.75 [0.61, 0.91]**	
Heterogeneity: Chi² = 1.21, df = 1 (P = 0.27); I² = 17%							
Test for overall effect: Z = 2.91 (P = 0.004)							
**Total (95% CI)**			**1127**	**1118**	**100.0%**	**0.81 [0.69, 0.95]**	
Heterogeneity: Chi² = 3.75, df = 3 (P = 0.29); I² = 20%							
Test for overall effect: Z = 2.53 (P = 0.01)							
Test for subgroup differences: Chi² = 2.13, df = 1 (P = 0.14), I² = 53.0%							

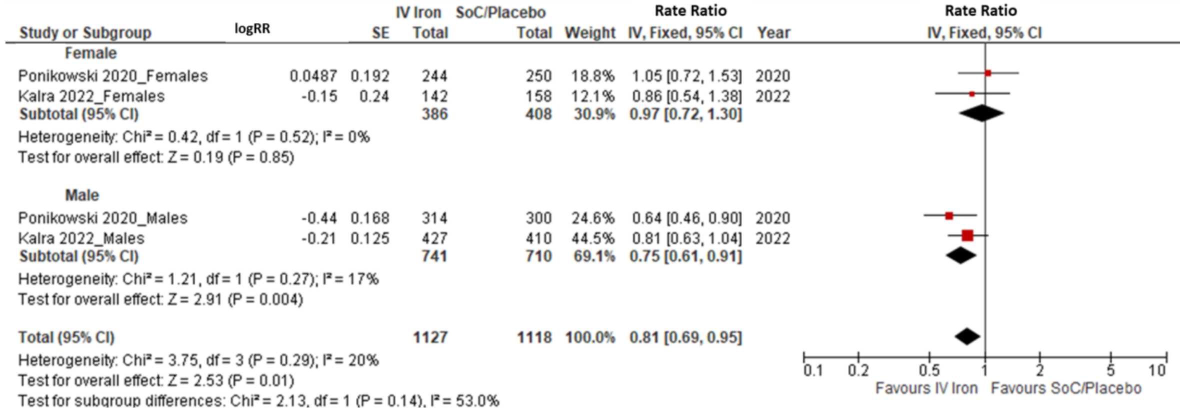

C

Fixed Effects: Recurrent HHF and CV death by NYHA class

Study or Subgroup	logRR	SE	IV Iron Total	SoC/Placebo Total	Weight	Rate Ratio IV, Fixed, 95% CI	Year
**NYHA II**							
Ponikowski 2020_NYHA II	-0.446	0.19	255	240	19.4%	0.64 [0.44, 0.93]	2020
Kalra 2022_NYHA II	-0.301	0.171	328	320	23.9%	0.74 [0.53, 1.03]	2022
**Subtotal (95% CI)**			**583**	**560**	**43.3%**	**0.69 [0.54, 0.89]**	
Heterogeneity: Chi² = 0.32, df = 1 (P = 0.57); I² = 0%							
Test for overall effect: Z = 2.88 (P = 0.004)							
**NYHA III or III/IV**							
Ponikowski 2020_NYHA III	-0.083	0.176	272	277	22.6%	0.92 [0.65, 1.30]	2020
Kalra 2022_NYHA III/IV	-0.094	0.143	241	248	34.2%	0.91 [0.69, 1.20]	2022
**Subtotal (95% CI)**			**513**	**525**	**56.7%**	**0.91 [0.74, 1.14]**	
Heterogeneity: Chi² = 0.00, df = 1 (P = 0.96); I² = 0%							
Test for overall effect: Z = 0.81 (P = 0.42)							
**Total (95% CI)**			**1096**	**1085**	**100.0%**	**0.81 [0.69, 0.96]**	
Heterogeneity: Chi² = 3.00, df = 3 (P = 0.39); I² = 0%							
Test for overall effect: Z = 2.50 (P = 0.01)							
Test for subgroup differences: Chi² = 2.68, df = 1 (P = 0.10), I² = 62.7%							

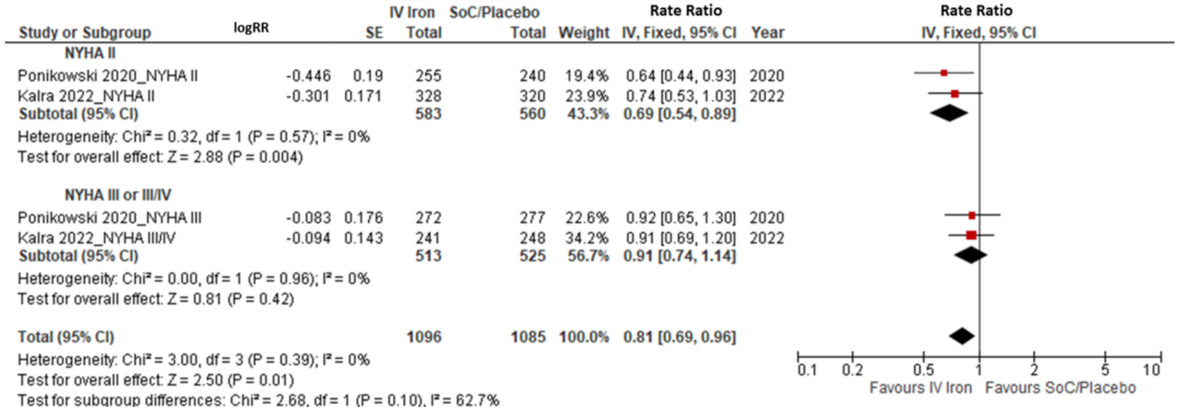

B

Fixed Effects: Recurrent HHF and CV death by tertiles of age

Study or Subgroup	logRR	SE	IV Iron Total	SoC/Placebo Total	Weight	Rate Ratio IV, Fixed, 95% CI	Year
**Tertile 1**							
Ponikowski 2020_Age_tertile 1	-0.673	0.228	166	174	12.7%	0.51 [0.33, 0.80]	2020
Kalra 2022_Age_tertile 1	-0.287	0.213	191	187	14.6%	0.75 [0.49, 1.14]	2022
**Subtotal (95% CI)**			**357**	**361**	**27.3%**	**0.63 [0.46, 0.85]**	
Heterogeneity: Chi² = 1.53, df = 1 (P = 0.22); I² = 35%							
Test for overall effect: Z = 3.00 (P = 0.003)							
**Tertile 2**							
Ponikowski 2020_Age_tertile 2	-0.15	0.212	196	175	14.7%	0.86 [0.57, 1.30]	2020
Kalra 2022_Age_tertile 2	0.086	0.182	185	194	20.0%	1.09 [0.76, 1.56]	2022
**Subtotal (95% CI)**			**381**	**369**	**34.7%**	**0.99 [0.75, 1.29]**	
Heterogeneity: Chi² = 0.71, df = 1 (P = 0.40); I² = 0%							
Test for overall effect: Z = 0.10 (P = 0.92)							
**Tertile 3**							
Ponikowski 2020_Age_tertile 3	0.029	0.213	196	201	14.6%	1.03 [0.68, 1.56]	2020
Kalra 2022_Age_tertile 3	-0.385	0.168	193	187	23.4%	0.68 [0.49, 0.95]	2022
**Subtotal (95% CI)**			**389**	**388**	**38.0%**	**0.80 [0.62, 1.03]**	
Heterogeneity: Chi² = 2.33, df = 1 (P = 0.13); I² = 57%							
Test for overall effect: Z = 1.72 (P = 0.09)							
**Total (95% CI)**			**1127**	**1118**	**100.0%**	**0.80 [0.69, 0.94]**	
Heterogeneity: Chi² = 9.31, df = 5 (P = 0.10); I² = 46%							
Test for overall effect: Z = 2.69 (P = 0.007)							
Test for subgroup differences: Chi² = 4.74, df = 2 (P = 0.09), I² = 57.8%							

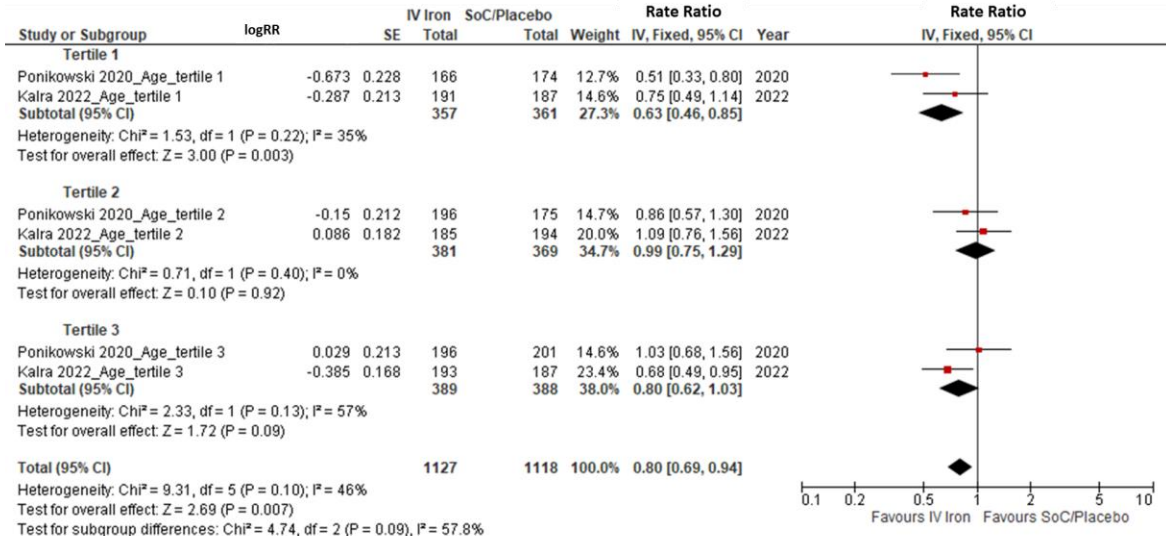

D

Fixed Effects: Recurrent HHF and CV death by tertiles of eGFR

Study or Subgroup	logRR	SE	IV Iron Total	SoC/Placebo Total	Weight	Rate Ratio IV, Fixed, 95% CI	Year
**eGFR tertile 1**							
Ponikowski 2020_eGFR tertile 1	-0.274	0.214	157	165	16.0%	0.76 [0.50, 1.16]	2020
Kalra 2022_eGFR tertile 1	-0.34	0.163	188	190	27.6%	0.71 [0.52, 0.98]	2022
**Subtotal (95% CI)**			**345**	**355**	**43.7%**	**0.73 [0.57, 0.94]**	
Heterogeneity: Chi² = 0.06, df = 1 (P = 0.81); I² = 0%							
Test for overall effect: Z = 2.44 (P = 0.01)							
**eGFR tertile 2**							
Ponikowski 2020_eGFR tertile 2	-0.274	0.237	157	165	13.1%	0.76 [0.48, 1.21]	2020
Kalra 2022_eGFR tertile 2	-0.139	0.195	191	188	19.3%	0.87 [0.59, 1.28]	2022
**Subtotal (95% CI)**			**348**	**353**	**32.4%**	**0.82 [0.61, 1.11]**	
Heterogeneity: Chi² = 0.19, df = 1 (P = 0.66); I² = 0%							
Test for overall effect: Z = 1.29 (P = 0.20)							
**eGFR tertile 3**							
Ponikowski 2020_eGFR tertile 3	-0.371	0.25	164	159	11.7%	0.69 [0.42, 1.13]	2020
Kalra 2022_eGFR tertile 3	-0.094	0.245	190	190	12.2%	0.91 [0.56, 1.47]	2022
**Subtotal (95% CI)**			**354**	**349**	**24.0%**	**0.79 [0.56, 1.12]**	
Heterogeneity: Chi² = 0.63, df = 1 (P = 0.43); I² = 0%							
Test for overall effect: Z = 1.31 (P = 0.19)							
**Total (95% CI)**			**1047**	**1057**	**100.0%**	**0.77 [0.65, 0.92]**	
Heterogeneity: Chi² = 1.29, df = 5 (P = 0.94); I² = 0%							
Test for overall effect: Z = 2.98 (P = 0.003)							
Test for subgroup differences: Chi² = 0.41, df = 2 (P = 0.82), I² = 0%							

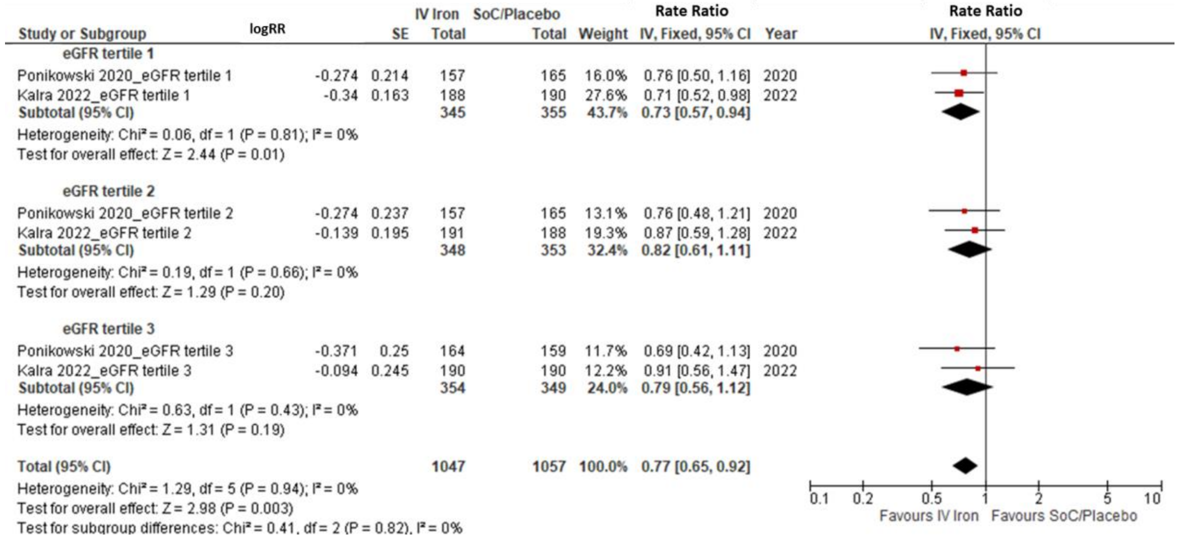

Supplementary Figure S6

A

Fixed Effects: Recurrent HHF and CV death by tertiles of Hb

Study or Subgroup	logRR	SE	IV Iron Total	SoC/Placebo Total	Weight	Rate Ratio IV, Fixed, 95% CI	Year
**15.1.1 Hb tertile 1**							
Anker IPD 2018_Hb_tertile 1	-0.653	0.436	166	104	3.3%	0.52 [0.22, 1.22]	2018
Ponikowski 2020_Hb_1	-0.03	0.193	228	236	16.8%	0.97 [0.66, 1.42]	2020
Kalra 2022_Hb_tertile 1	-0.235	0.172	179	186	21.1%	0.79 [0.56, 1.11]	2022
**Subtotal (95% CI)**			**573**	**526**	**41.2%**	**0.83 [0.65, 1.06]**	
Heterogeneity: Chi² = 1.88, df = 2 (P = 0.39); I² = 0%							
Test for overall effect: Z = 1.50 (P = 0.13)							
**15.1.2 Hb tertile 2**							
Anker IPD 2018_Hb_tertile_2	-0.562	0.422	164	105	3.5%	0.57 [0.25, 1.30]	2020
Kalra 2022_Hb_tertile 2	-0.139	0.216	196	175	13.4%	0.87 [0.57, 1.33]	2022
**Subtotal (95% CI)**			**360**	**280**	**16.9%**	**0.80 [0.55, 1.16]**	
Heterogeneity: Chi² = 0.80, df = 1 (P = 0.37); I² = 0%							
Test for overall effect: Z = 1.18 (P = 0.24)							
**15.1.3 Hb tertile 3**							
Anker IPD_2018_Hb_tertile 3	-0.673	0.408	174	126	3.8%	0.51 [0.23, 1.14]	2018
Ponikowski 2020_Hb_2	-0.4	0.168	329	314	22.2%	0.67 [0.48, 0.93]	2020
Kalra 2022_Hb_tertile 3	-0.174	0.198	194	207	16.0%	0.84 [0.57, 1.24]	2022
**Subtotal (95% CI)**			**697**	**647**	**41.9%**	**0.71 [0.56, 0.91]**	
Heterogeneity: Chi² = 1.50, df = 2 (P = 0.47); I² = 0%							
Test for overall effect: Z = 2.77 (P = 0.006)							
**Total (95% CI)**			**1630**	**1453**	**100.0%**	**0.77 [0.66, 0.90]**	
Heterogeneity: Chi² = 4.99, df = 7 (P = 0.66); I² = 0%							
Test for overall effect: Z = 3.24 (P = 0.001)							
Test for subgroup differences: Chi² = 0.81, df = 2 (P = 0.67), I² = 0%							

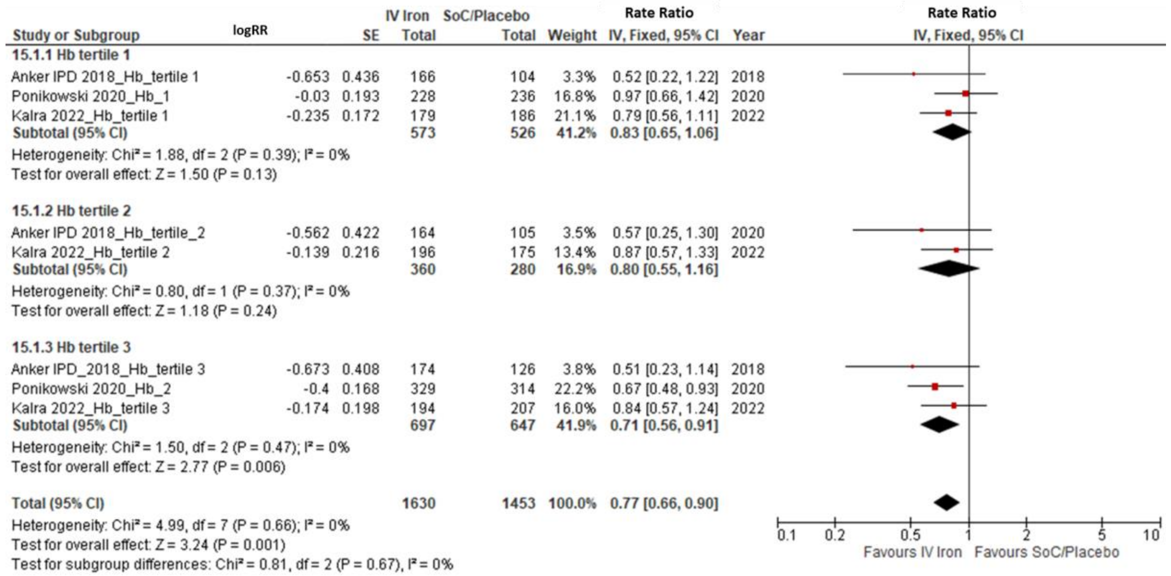

B

Fixed Effects: Recurrent HHF and CV death by TSAT < or ≥20%

Study or Subgroup	logRR	SE	IV Iron Total	SoC/Placebo Total	Weight	Rate Ratio IV, Fixed, 95% CI	Year
**17.1.1 TSAT <20%**							
Anker IPD 2018_TSAT tertile 1	-1.203	0.353	174	105	4.4%	0.30 [0.15, 0.60]	2018
Anker IPD 2018_TSAT tertile 2	-0.415	0.431	166	116	3.0%	0.66 [0.28, 1.54]	2018
Ponikowski 2020_TSAT_20_1	-0.267	0.136	457	469	30.0%	0.77 [0.59, 1.00]	2020
Kalra 2022_TSAT_20_1	-0.223	0.125	413	428	35.5%	0.80 [0.63, 1.02]	2022
**Subtotal (95% CI)**			**1210**	**1118**	**72.9%**	**0.73 [0.62, 0.87]**	
Heterogeneity: Chi² = 7.04, df = 3 (P = 0.07); I² = 57%							
Test for overall effect: Z = 3.54 (P = 0.0004)							
**17.1.2 TSAT >=20%**							
Anker IPD 2018_TSAT tertile 3	0.636	0.577	162	114	1.7%	1.89 [0.61, 5.85]	2018
Ponikowski 2020_TSAT_20_2	-0.116	0.308	97	78	5.8%	0.89 [0.49, 1.63]	2020
Kalra 2022_TSAT_20_2	-0.04	0.168	141	128	19.6%	0.96 [0.69, 1.34]	2022
**Subtotal (95% CI)**			**400**	**320**	**27.1%**	**0.99 [0.74, 1.30]**	
Heterogeneity: Chi² = 1.40, df = 2 (P = 0.50); I² = 0%							
Test for overall effect: Z = 0.10 (P = 0.92)							
**Total (95% CI)**			**1610**	**1438**	**100.0%**	**0.80 [0.69, 0.92]**	
Heterogeneity: Chi² = 11.53, df = 6 (P = 0.07); I² = 48%							
Test for overall effect: Z = 3.08 (P = 0.002)							
Test for subgroup differences: Chi² = 3.08, df = 1 (P = 0.08), I² = 67.6%							

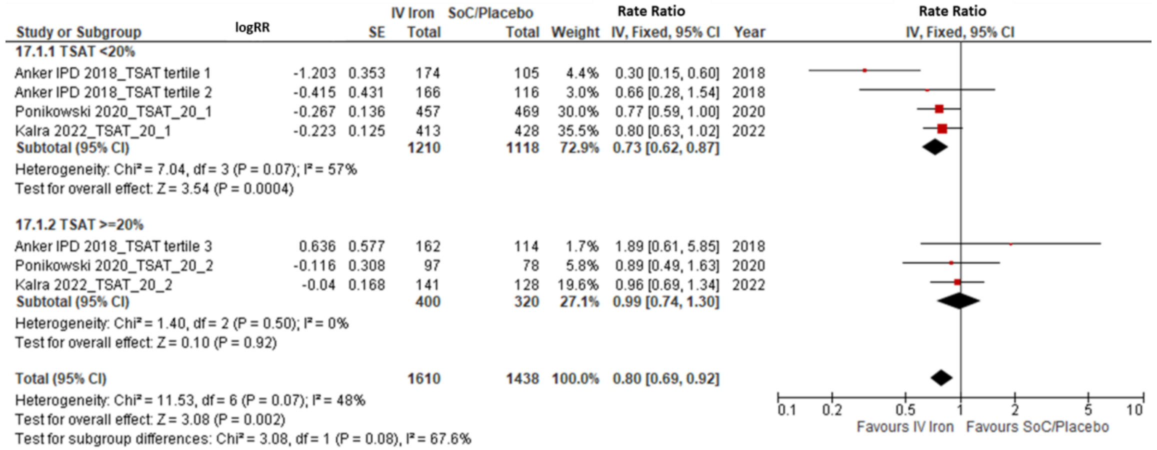

C

Fixed Effects: Recurrent HHF and CV death by tertiles of Ferritin

Study or Subgroup	logRR	SE	IV Iron Total	SoC/Placebo Total	Weight	Rate Ratio IV, Fixed, 95% CI	Year
**16.1.1 Ferritin tertile 1**							
Anker IPD 2018_Ferritin tertile 1	-0.4	0.523	178	96	2.5%	0.67 [0.24, 1.87]	2018
Ponikowski 2020_Ferritin_tertile 1	-0.301	0.222	180	188	14.1%	0.74 [0.48, 1.14]	2020
Kalra 2022_Ferritin tertile 1	-0.22	0.219	189	183	14.5%	0.80 [0.52, 1.23]	2022
**Subtotal (95% CI)**			**547**	**467**	**31.2%**	**0.76 [0.57, 1.02]**	
Heterogeneity: Chi² = 0.13, df = 2 (P = 0.94); I² = 0%							
Test for overall effect: Z = 1.82 (P = 0.07)							
**16.1.2 Ferritin tertile 2**							
Anker IPD 2018_Ferritin tertile 2	-0.494	0.395	162	119	4.5%	0.61 [0.28, 1.32]	2018
Ponikowski 2020_Ferritin tertile 2	-0.385	0.211	203	167	15.6%	0.68 [0.45, 1.03]	2020
Kalra 2022_Ferritin tertile 2	-0.072	0.176	184	193	22.5%	0.93 [0.66, 1.31]	2022
**Subtotal (95% CI)**			**549**	**479**	**42.6%**	**0.79 [0.62, 1.02]**	
Heterogeneity: Chi² = 1.79, df = 2 (P = 0.41); I² = 0%							
Test for overall effect: Z = 1.81 (P = 0.07)							
**16.1.3 Ferritin tertile 3**							
Anker IPD 2018_Ferritin tertile 3	-0.798	0.384	164	120	4.7%	0.45 [0.21, 0.96]	2018
Ponikowski 2020_Ferritin tertile 3	-0.072	0.851	174	195	1.0%	0.93 [0.18, 4.93]	2020
Kalra 2022_Ferritin tertile 3	-0.314	0.184	196	190	20.6%	0.73 [0.51, 1.05]	2022
**Subtotal (95% CI)**			**534**	**505**	**26.2%**	**0.68 [0.49, 0.93]**	
Heterogeneity: Chi² = 1.44, df = 2 (P = 0.49); I² = 0%							
Test for overall effect: Z = 2.41 (P = 0.02)							
**Total (95% CI)**			**1630**	**1451**	**100.0%**	**0.75 [0.64, 0.88]**	
Heterogeneity: Chi² = 3.98, df = 8 (P = 0.86); I² = 0%							
Test for overall effect: Z = 3.43 (P = 0.0006)							
Test for subgroup differences: Chi² = 0.62, df = 2 (P = 0.73), I² = 0%							

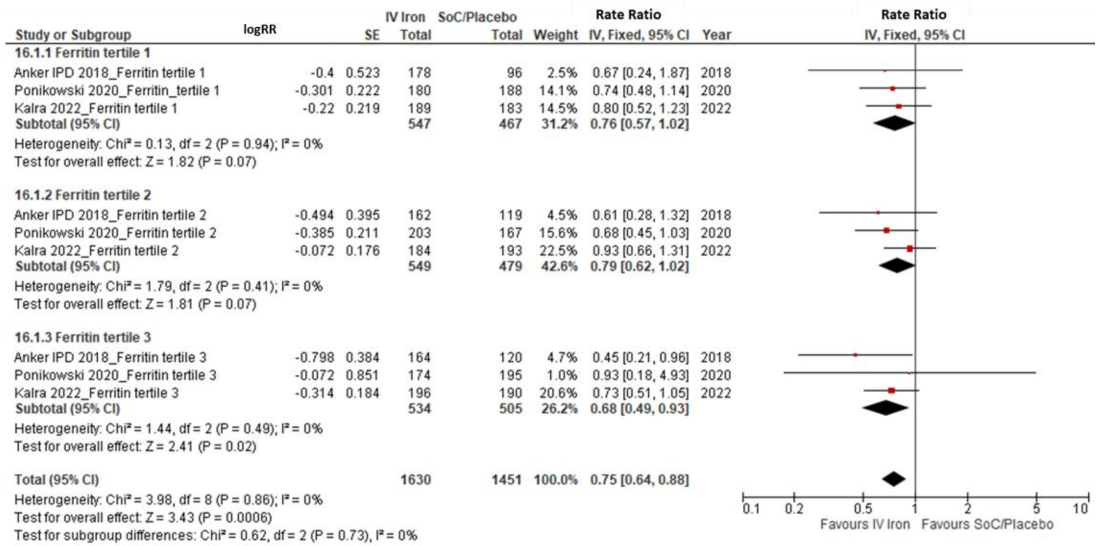

D

Fixed Effects: Recurrent HHF and CV death by tertiles of TSAT

Study or Subgroup	logRR	SE	IV Iron Total	SoC/Placebo Total	Weight	Rate Ratio IV, Fixed, 95% CI	Year
**18.1.1 TSAT tertile 1**							
Anker IPD 2018_TSAT tertile 1	-1.203	0.353	174	105	5.1%	0.30 [0.15, 0.60]	2018
Ponikowski 2020_TSAT tertile 1	-0.301	0.238	179	187	11.3%	0.74 [0.46, 1.18]	2020
Kalra 2022_TSAT tertile 1	-0.162	0.187	163	169	18.3%	0.85 [0.59, 1.23]	2022
**Subtotal (95% CI)**			**516**	**461**	**34.8%**	**0.70 [0.53, 0.91]**	
Heterogeneity: Chi² = 6.89, df = 2 (P = 0.03); I² = 71%							
Test for overall effect: Z = 2.66 (P = 0.008)							
**18.1.2 TSAT tertile 2**							
Anker IPD 2018_TSAT tertile 2	-0.415	0.431	166	116	3.4%	0.66 [0.28, 1.54]	2018
Ponikowski 2020_TSAT tertile 2	-0.371	0.216	181	187	13.7%	0.69 [0.45, 1.05]	2020
Kalra 2022_TSAT tertile 2	-0.083	0.208	188	183	14.8%	0.92 [0.61, 1.38]	2022
**Subtotal (95% CI)**			**535**	**486**	**32.0%**	**0.78 [0.59, 1.04]**	
Heterogeneity: Chi² = 1.10, df = 2 (P = 0.58); I² = 0%							
Test for overall effect: Z = 1.71 (P = 0.09)							
**18.1.3 TSAT tertile 3**							
Anker IPD 2018_TSAT tertile 3	0.636	0.577	162	114	1.9%	1.89 [0.61, 5.85]	2018
Ponikowski 2020_TSAT tertile 3	-0.072	0.217	194	173	13.6%	0.93 [0.61, 1.42]	2020
Kalra 2022_TSAT tertile 3	-0.261	0.19	203	205	17.7%	0.77 [0.53, 1.12]	2022
**Subtotal (95% CI)**			**559**	**492**	**33.3%**	**0.88 [0.67, 1.15]**	
Heterogeneity: Chi² = 2.31, df = 2 (P = 0.32); I² = 13%							
Test for overall effect: Z = 0.95 (P = 0.34)							
**Total (95% CI)**			**1610**	**1439**	**100.0%**	**0.78 [0.67, 0.91]**	
Heterogeneity: Chi² = 11.69, df = 8 (P = 0.17); I² = 32%							
Test for overall effect: Z = 3.09 (P = 0.002)							
Test for subgroup differences: Chi² = 1.40, df = 2 (P = 0.50), I² = 0%							

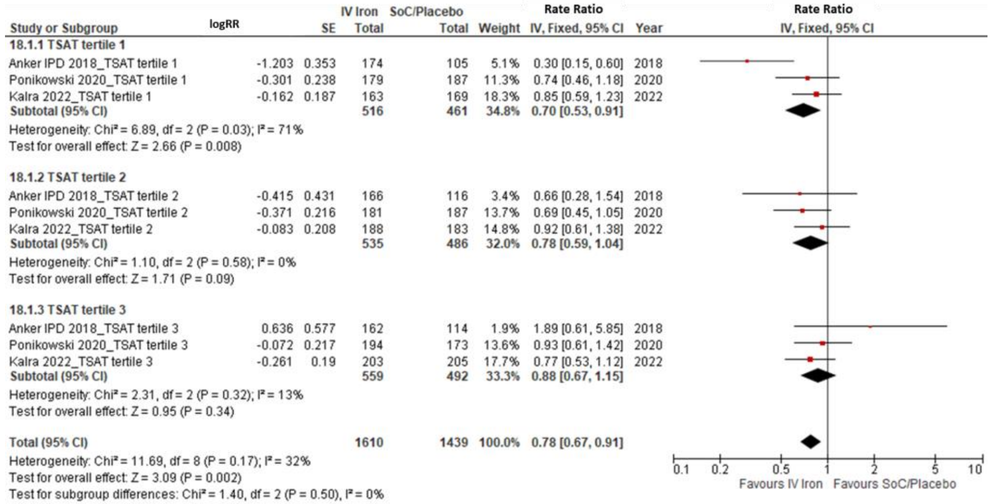

Supplementary Figure S7

A

Fixed Effects: Recurrent HHF and CV death by heart failure diagnosis (new vs persistent)

Study or Subgroup	logRR	SE	IV Iron Total	SoC/Placebo Total	Weight	Rate Ratio IV, Fixed, 95% CI	Year
**De-novo Heart Failure**							
Ponikowski 2020_HF_de novo	0.198	0.288	153	165	8.1%	1.22 [0.69, 2.14]	2020
Kalra 2022_HF_de novo	-0.248	0.469	51	64	3.0%	0.78 [0.31, 1.96]	2022
**Subtotal (95% CI)**			**204**	**229**	**11.1%**	**1.08 [0.67, 1.75]**	
Heterogeneity: Chi² = 0.66, df = 1 (P = 0.42); I² = 0%							
Test for overall effect: Z = 0.31 (P = 0.76)							
**Pre-existent Heart Failure**							
Ponikowski 2020_HF_pre-existent	-0.328	0.139	405	385	34.7%	0.72 [0.55, 0.95]	2020
Kalra 2022_HF_less than 1 year	-0.21	0.243	151	146	11.3%	0.81 [0.50, 1.31]	2022
Kalra 2022_HF_over 1 year	-0.223	0.125	367	358	42.9%	0.80 [0.63, 1.02]	2022
**Subtotal (95% CI)**			**923**	**889**	**88.9%**	**0.77 [0.65, 0.91]**	
Heterogeneity: Chi² = 0.37, df = 2 (P = 0.83); I² = 0%							
Test for overall effect: Z = 3.02 (P = 0.003)							
**Total (95% CI)**			**1127**	**1118**	**100.0%**	**0.80 [0.68, 0.94]**	
Heterogeneity: Chi² = 2.71, df = 4 (P = 0.61); I² = 0%							
Test for overall effect: Z = 2.75 (P = 0.006)							
Test for subgroup differences: Chi² = 1.69, df = 1 (P = 0.19), I² = 40.7%							

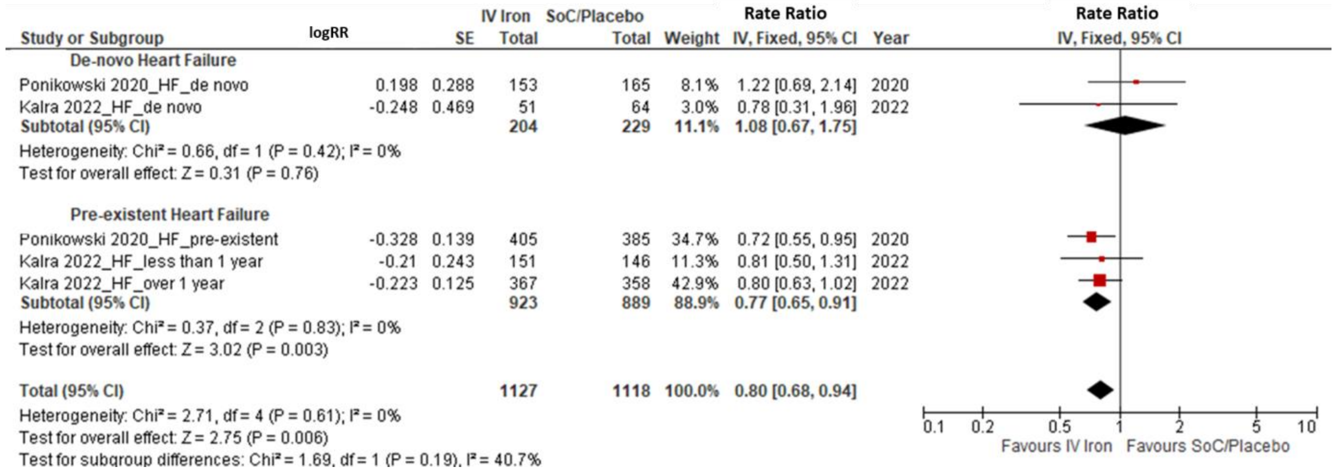

B

Fixed Effects: Recurrent HHF and CV death by heart failure aetiology (ischaemic vs non)

Study or Subgroup	logRR	SE	IV Iron Total	SoC/Placebo Total	Weight	Rate Ratio IV, Fixed, 95% CI	Year
**IHD**							
Ponikowski 2020_IHD	-0.15	0.065	265	257	66.8%	0.86 [0.76, 0.98]	2020
Kalra 2022_IHD	-0.287	0.132	331	316	16.2%	0.75 [0.58, 0.97]	2022
**Subtotal (95% CI)**			**596**	**573**	**82.9%**	**0.84 [0.75, 0.94]**	
Heterogeneity: Chi² = 0.87, df = 1 (P = 0.35); I² = 0%							
Test for overall effect: Z = 3.03 (P = 0.002)							
**No IHD**							
Ponikowski 2020_no IHD	0.104	0.186	282	275	8.2%	1.11 [0.77, 1.60]	2020
Kalra 2022_no IHD	-0.139	0.178	238	252	8.9%	0.87 [0.61, 1.23]	2022
**Subtotal (95% CI)**			**520**	**527**	**17.1%**	**0.98 [0.76, 1.26]**	
Heterogeneity: Chi² = 0.89, df = 1 (P = 0.35); I² = 0%							
Test for overall effect: Z = 0.18 (P = 0.86)							
**Total (95% CI)**			**1116**	**1100**	**100.0%**	**0.86 [0.78, 0.95]**	
Heterogeneity: Chi² = 2.95, df = 3 (P = 0.40); I² = 0%							
Test for overall effect: Z = 2.83 (P = 0.005)							
Test for subgroup differences: Chi² = 1.19, df = 1 (P = 0.28), I² = 15.8%							

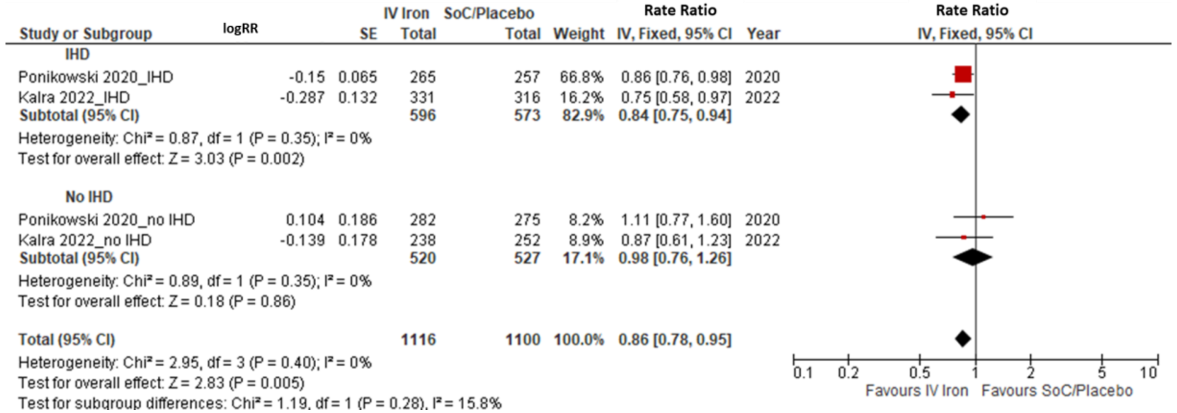

Supplementary Figure S8

Unique ID	Experimental	Comparator	Outcome	Weight	D1	D2	D3	D4	D5	Overall
Kalra 2022	IV Iron	SoC	NA	1	+	+	+	+	+	+
Ponikowski 2020	IV iron	SoC	NA	1	+	+	+	+	+	+
Martens 2021	IV Iron	Placebo	NA	1	+	+	+	+	+	+
Dhoot 2020	IV Iron	SoC	NA	1	+	!	+	!	!	!
Yeo 2018	IV Iron	Placebo	NA	1	+	+	+	+	+	+
van Velduisen 2017	IV Iron	SoC	NA	1	+	+	+	+	+	+
Ponikowski 2014	IV Iron	Placebo	NA	1	+	+	+	+	+	+
Anker 2009	IV Iron	Placebo	NA	1	+	+	+	+	+	+
Okonko 2008	IV Iron	SoC	NA	1	+	+	+	!	+	!
Toblli 2007	IV Iron	Placebo	NA	1	+	+	+	+	!	!

\+ Low risk
! Some concerns
\- High risk

D1 Randomisation process
D2 Deviations from the intended interventions
D3 Missing outcome data
D4 Measurement of the outcome
D5 Selection of the reported result
